# A toolbox for manipulating the genome of the major goat pathogen, *Mycoplasma capricolum* subsp. *capripneumoniae*

**DOI:** 10.1099/mic.0.001423

**Published:** 2024-01-09

**Authors:** Géraldine Gourgues, Lucía Manso-Silván, Catherine Chamberland, Pascal Sirand-Pugnet, François Thiaucourt, Alain Blanchard, Vincent Baby, Carole Lartigue

**Affiliations:** ^1^​ Université de Bordeaux, INRAE, BFP, UMR 1332, F-33140 Villenave d'Ornon, France; ^2^​ CIRAD, UMR ASTRE, F-34398, Montpellier, France; ^3^​ ASTRE, Université de Montpellier, CIRAD, INRAE, F-34398, Montpellier, France; ^4^​ Université de Sherbrooke, Département de biologie, Sherbrooke, Québec, J1K 2R1, Canada; ^5^​ Université de Montréal, Faculté de médecine vétérinaire, Saint-Hyacinthe, Québec, J2S 2M2, Canada

**Keywords:** Cre–lox recombination system, in-yeast genome cloning/engineering, *Mycoplasma capricolum* subsp. *capripneumoniae*, *oriC* plasmid, S41 serine protease, whole-genome transplantation

## Abstract

*Mycoplasma capricolum* subspecies *capripneumoniae* (*Mccp*) is the causative agent of contagious caprine pleuropneumonia (CCPP), a devastating disease listed by the World Organisation for Animal Health (WOAH) as a notifiable disease and threatening goat production in Africa and Asia. Although a few commercial inactivated vaccines are available, they do not comply with WOAH standards and there are serious doubts regarding their efficacy. One of the limiting factors to comprehend the molecular pathogenesis of CCPP and develop improved vaccines has been the lack of tools for *Mccp* genome engineering. In this work, key synthetic biology techniques recently developed for closely related mycoplasmas were adapted to *Mccp*. CReasPy-Cloning was used to simultaneously clone and engineer the *Mccp* genome in yeast, prior to whole-genome transplantation into *M. capricolum* subsp. *capricolum* recipient cells. This approach was used to knock out an S41 serine protease gene recently identified as a potential virulence factor, leading to the generation of the first site-specific *Mccp* mutants. The Cre–lox recombination system was then applied to remove all DNA sequences added during genome engineering. Finally, the resulting unmarked S41 serine protease mutants were validated by whole-genome sequencing and their non-caseinolytic phenotype was confirmed by casein digestion assay on milk agar. The synthetic biology tools that have been successfully implemented in *Mccp* allow the addition and removal of genes and other genetic features for the construction of seamless targeted mutants at ease, which will pave the way for both the identification of key pathogenicity determinants of *Mccp* and the rational design of novel, improved vaccines for the control of CCPP.

## Data Summary

The data supporting the findings of this study are available within the paper and its Supplementary Material. The sequences from this study are available from the National Center for Biotechnology Information (NCBI) under Sequence Read Archive (SRA) accession nos SAMN32543038, SAMN32543039 and SAMN32543040.

**Impact Statement**

Due to its high morbidity/mortality and potential to spread rapidly across borders, inducing significant socio-economic damage, contagious caprine pleuropneumonia (CCPP) is included in the list of notifiable diseases of the World Organisation for Animal Health. Study of its causal agent, Mycoplasma capricolum subsp. *capripneumoniae* (*Mccp*), is difficult due both to its fastidious nature and the lack of dedicated genetic tools to manipulate its genome. Therefore, the control and prevention of CCPP have been limited and the geographical range of the disease is expanding. In the present study, using methods derived from synthetic biology, we have developed a new approach allowing the production of seamless targeted *Mccp* mutants. This new toolbox should allow us to better understand the functional genomics of *Mccp* and pave the way for the production of rationally designed vaccines for the control of CCPP.

## Introduction

*Mycoplasma capricolum* subspecies *capripneumoniae* (*Mccp*) is the aetiological agent of contagious caprine pleuropneumonia (CCPP), a severe respiratory disease of goats characterized by high morbidity and mortality, as well as the potential to spread across borders, reaching epizootic proportions. CCPP has thus been included in the list of notifiable diseases of the World Organisation for Animal Health (WOAH; previously the OIE; https://www.woah.org). Although its exact distribution is not well known, CCPP is endemic in arid and semiarid regions of Africa, the Middle East and Asia, where it poses a major threat to goat rearing and to the livelihood of small ruminant smallholders [1–3]. The introduction of the disease into the Thrace region of Turkey and Mauritius, where *Mccp* was isolated in 2004 and 2009, respectively [4, 5], shows that CCPP represents a serious risk for disease-free areas, including Europe. Furthermore, CCPP was also shown to affect a variety of captive and free-ranging wild ungulates in the Middle East [6–8] and Tibet [9], threatening the conservation of endangered species and raising concerns for zoos and game resorts.

In regions where CCPP is endemic, antibiotic treatments are often the only means of control, since vaccines are not widely available and animal movement control and stamping-out policies are both impractical and unacceptable for socio-economic reasons [10]. Although antibiotherapy can significantly reduce the mortality and lesions, it does not clear the infection [11], so treated animals may act as asymptomatic *Mccp* carriers. Furthermore, treatments are often applied under suboptimal conditions in the field, increasing the risk of antimicrobial resistance, which is one of the greatest health challenges of our times [12] (https://amr.tghn.org/resources/one-health/ and https://www.fao.org/fao-who-codexalimentarius/about-codex/en/). For these reasons, medical prophylaxis based on the use of vaccines is the preferred method to reduce the prevalence and limit the expansion of CCPP in these regions [1].

The only CCPP vaccine currently prescribed by the WOAH was developed over 30 years ago and consists of *Mccp* whole-cell antigens inactivated and adjuvanted with saponin [13]. According to WOAH guidelines [3], the antigen and saponin content in these vaccines is extremely high (i.e*.* 0.15 and 3 mg per dose, respectively). However, the fastidious nature of *Mccp*, which presents slow growth and requires rich and expensive culture media, makes antigen production very costly, while the high dose of crude saponin required to induce good protection is incompatible with current safety standards. Although a few commercial CCPP vaccines are currently available, a recent study using mass spectrometry to analyse their protein composition revealed that they were composed of very small quantities of specific mycoplasma antigen and high amounts of residual proteins originating from the production medium [14]. These results proved that commercially available vaccines do not meet the WOAH standards, casting serious doubts on their quality and highlighting the urgent need for improvement.

The rational design of effective vaccines inducing protective and long-lasting host immunity generally requires a good knowledge of the molecular mechanisms and factors underpinning pathogenesis and protection [15]. The paucity of both genetic tools and efficient transformation protocols for use in mycoplasmas have long hampered the implementation of functional genomics studies, which, in turn, has significantly delayed our understanding of mycoplasma diseases and the development of improved vaccines [15]. The first examples of targeted gene inactivation appeared in the early 2000s and were based on the use of *oriC* artificial plasmids. This approach allowed the characterization of the first targeted mutants in several species of mycoplasmas [16–18], but turned out to be tedious, as the integration of the *oriC* replicative plasmid at the target gene generally required extensive passaging. Nevertheless, those tools proved to be valuable for both heterologous protein expression and gene complementation studies [16, 19, 20].

Since those early experiments, more and more sophisticated methods, allowing both small-scale and large-scale genome modifications, have emerged to such an extent that today almost all kinds of genetic modifications are within reach, at least for some mycoplasma species. These methods can be classified into two categories: those that allow the modification of mycoplasma genomes directly inside the cell (‘in-mycoplasma’ methods) and those that use the yeast *Saccharomyces cerevisiae* as a temporary host to edit the imported genomes. The latter, a synthetic biology approach, is the most powerful, since it makes it possible to perform gene insertions, deletions and replacements, point mutations, as well as heterologous gene expression at any desired locus and, in most cases, without leaving any trace or scar [21–27]. However, this approach is only currently available for six mycoplasma species [24, 28, 29]. Moreover, it is less straightforward than in-bacteria editing strategies, since the entire target genome (native or synthetic) must first be cloned in yeast to be modified using yeast genetic tools, and then back-transplanted into a recipient bacterium to produce an engineered mycoplasma cell. For these reasons, having access to a set of tools that can be used to directly manipulate mycoplasma genomes is extremely convenient. The mycoplasma genetic toolbox has recently diversified [30]. In addition to few transposons [31–37] and the *oriC* plasmids mentioned above, it now also includes the exogenous recombination RecET-like system from the *Bacillus subtilis* phage SspI [38–40], CRISPR/Cas editing tools from *Mycoplasma gallisepticum* [41, 42], the Cas9 base editor system [43], CRISPR interference (CRISPRi)-based transposons with *Streptococcus pyogenes* dCas9 [44, 45] and the Cre–lox system from the *Escherichia coli* phage P1 [39, 46, 47]. Despite a range of action that is rather restricted to small-scale modifications and some other limitations (e.g. the insertion of supernumerary DNA cassettes in the genome of interest), these tools make it possible to inactivate or silence genes (CRISPR/Cas-based tools, RecET and *OriC* plasmids) [39, 41–45], express heterologous genes (transposons and *OriC* plasmid) [16], or remove unwanted genes such as antibiotic resistance markers (Cre–lox) [39, 46, 47].

The technological leap that has taken place in recent years around the manipulation of microbial genomes and the development of genetic tools is such that we now have access to a panel of diversified and multifaceted tools that can meet most of our needs in terms of DNA engineering. This context prompted us to evaluate some of the new tools in *Mccp*, which has always been considered a particularly fastidious and genetically intractable species among mycoplasmas. Indeed, to date, no genetic tools or mutagenesis methods have been described in the literature for this species, which could lead one to believe that the production of targeted mutants is not possible for this major pathogen.

**Fig. 1.**
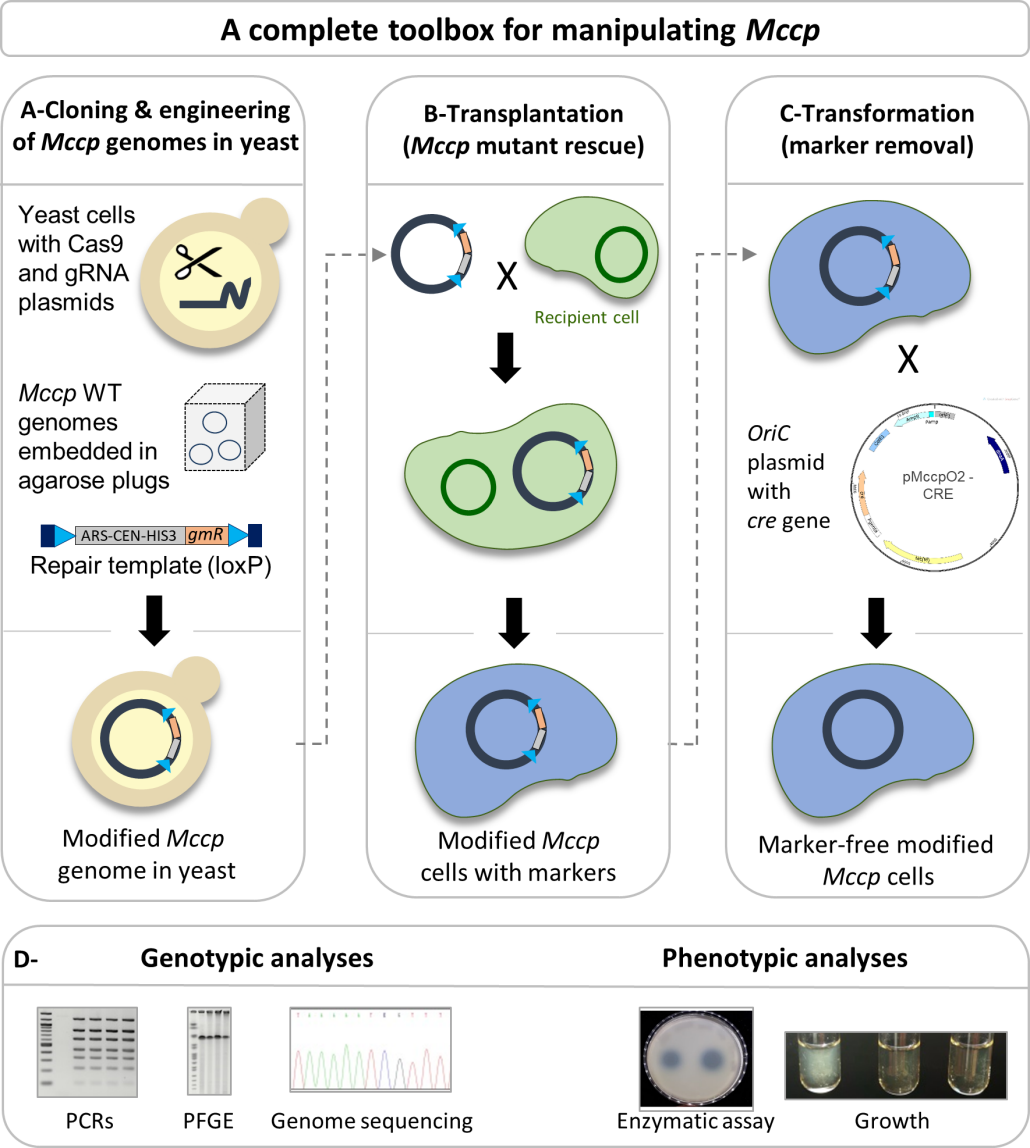
Global scheme representing the main steps conducted in this study. (a) *Mccp* WT donor genomes (isolated in agarose plugs) are introduced into yeast spheroplasts preloaded with Cas9 and gRNA expression plasmids along with a repair template. This latter linear DNA fragment contains the yeast elements ARSH4 (autonomous replicating sequence), CEN6 (centromere) and HIS3 (histidine auxotrophic marker) for maintenance and selection in yeast as well as a gentamicin resistance marker (*gmR*) for selection in *Mycoplasma*. The repair template is flanked by two 60 bp recombination arms homologous to each side of the target locus (dark blue rectangles, here the MCCP001_RS01320 locus). Two 34 pb loxP sites (light blue triangles) are added flanking the yeast elements and the antibiotic resistance marker. The Cas9 and gRNA plasmids carry the TRP1 and URA3 selection markers, respectively. Following transformation, the *Mccp* genome is cleaved by the Cas9/gRNA complex, and subsequently repaired by the yeast homologous recombination system, using the provided repair template. As a result, the bacterial genome, which includes the yeast elements in place of the targeted locus, can be propagated by the yeast as an artificial chromosome. (b) Newly modified *Mccp* genomes are then transplanted into *McapΔRE* recipient cells [27] to obtain the expected *Mccp* mutants. At this stage, mutants can be characterized, but they still harbour yeast elements and a gentamicin resistance marker. (c) To remove the additional genetic cassettes, mutants of interest are transformed with a replicative *oriC* plasmid carrying the *cre* recombinase gene along with a tetracycline resistance marker. Propagation of *Mccp* transformants in mycoplasma medium with tetracycline, followed by a few passages in medium without antibiotic, allows the recovery of *Mccp* mutants free of unwanted genetic markers. (d) At the different stages of implementation, transplants and transformants are characterized both at the genotypic and phenotypic levels.

In this study, we first demonstrate that the most flexible method available to date for whole-genome engineering of mycoplasmas, i.e. the in-yeast strategy, can be readily applied to *Mccp* and thus be used to produce site-specific *Mccp* mutants of interest. This was done by targeting a gene coding for an S41 serine protease with an assessable phenotype. Second, we show that some in-mycoplasma tools, more specifically *oriC* plasmids and the Cre–lox recombination system, are also functional in *Mccp* and can be used, following the in-yeast strategy, to generate seamless targeted mutants (Fig. 1). *Mccp* mutants were validated both at the genotypic and phenotypic level. This study definitely expands the possibilities for CCPP pathogenicity and vaccine research.

## Methods

### Bacterial strains, cultures conditions and commercial plasmids

*Mccp* strain 9231-Abomsa was isolated in Ethiopia in 1982 [48, 49]. The strain was grown at 37 °C in modified Hayflick’s medium [50] with 10 % additional horse serum (m-Hayflick medium), under a 5 % CO_2_ atmosphere. Gentamicin 300 µg ml^−1^, puromycin 8 µg ml^−1^ and tetracycline 0.5 µg ml^−1^ were used in selective conditions. *S. cerevisiae* strain VL6-48N (MATα, his3-Δ200, trp1-Δ1, ura3-Δ1, lys2, ade2-101, met14, cir^o^) was grown at 30 °C in YPDA medium or in synthetic defined (SD) medium depleted for one or several amino acids for selection (SD-His is, for example, a histidine-depleted SD medium). *E. coli* strains (NEB 10-beta Electrocompetent *E. coli* or NEB 5-alpha Competent *E. coli* High Efficiency), used as host strains for cloning experiments, were grown at 37 °C in Luria–Bertani (LB) medium. Ampicillin 100 µg ml^−1^ and tetracycline 5 µg ml^−1^ were used in selective conditions.

p414-TEF1p-Cas9-CYC1t plasmid, developed by DiCarlo *et al*. [51], was acquired from the Addgene repository (reference #43802) This plasmid is a centromeric plasmid with a CEN6/ARSH4 origin and contains the TRP1 selection marker.

p426-SNR52p-gRNA.CAN1.Y-SUP4t plasmid, developed by DiCarlo *et al*. [51], was acquired from the Addgene repository (reference #43803). It is a high-copy 2μ plasmid with a URA3 auxotrophic marker for selection. This plasmid was modified by Tsarmpopoulos *et al*. [25]: the CAN1.Y 20 bp spacer was replaced by an AarI cloning spacer in order to create a plasmid in which specific spacers can be easily inserted. This plasmid was named p426-SNR52p-gRNA.AarI.Y-SUP4t or pgRNA.AarI. All of the pgRNA constructed in this study was derived from the pgRNA.AarI (see section on plasmid construction).

MICs for the different antibiotics used for selection in mycoplasma cultures were determined according to the Clinical and Laboratory Standards Institute (CLSI) guidelines [52].

### Isolation of whole *Mccp* chromosomes in agarose plugs and transfer of mycoplasma genomes into yeast cells (CReasPy-Cloning method)

A 100 ml *Mccp* culture was prepared in m-Hayflick broth. When the medium turned turbid, chloramphenicol was added at a final concentration of 100 μg ml^−1^. Cells were then incubated for 1 h 45 min at 37 °C and harvested by centrifugation (7500 ***g*** for 15 min at 10 °C). The cell pellet was resuspended in 50 ml T/S buffer (10 mM Tris pH 6.5, 500 mM sucrose), centrifuged a second time in the same conditions and resuspended into ~500 µl T/S buffer. Cell suspension was mixed (vol/vol) with a 2 % low-melting-point agarose solution and treated following the instructions of the CHEF Mammalian Genomic DNA Plug kit (Biorad). This method yields agarose plugs that contain isolated and intact mycoplasma chromosomes. The quality of the *Mccp* strain 9231-Abomsa genomic DNA was assessed by digestion using 50 units of BssHII restriction enzymes (NEB) per plug followed by pulsed-field gel electrophoresis. Prior to yeast transformation, *Mccp* genomes were released by digestion of the agarose plugs with three units of β-agarase I (NEB) per plug. The DNA concentration in the digested plugs was measured using an Epoch Microplate Spectrophotometer coupled to a Take3 micro-volume plate (BioTek).

Yeast cells preloaded with p414-TEF1p-Cas9-CYC1t (pCas9) and p426-SNR52p-gRNA.AarI.Y-SUP4t (pgRNA.AarI) plasmids were transformed as previously described [53, 54]. Briefly, 100 µl of yeast spheroplasts were mixed with ~2 µg of newly isolated *Mccp* genomic DNA and 300 ng of recombination template made of the yeast elements (ARSH4, CEN6 and the auxotrophic marker HIS3), the *aacA-aphD* gentamicin resistance marker and 60 bp arms identical to the sequences flanking the MCCP001_RS01320 target gene in the *Mccp* genome. For this study, two recombination templates were produced. The first one was PCR-amplified from the pMT85P*genta*-PS*lacZ*-PRS313 plasmid (Fig. S1, available in the online version of this article) using the primer pairs MccpPeptS41pMT85gentPRS-F/MccpPeptS41pMT85gentPRS-F. The second one was produced with the primer pairs MccpPeptS41LOX66pMT85gentPRS-F/MccpPeptS41LOX71pMT85gentPRS-R in order to include loxP sites [55] in the amplicon (Fig. S2). After transformation, cells were selected on SD-His solid agar plates containing 1 M of sorbitol for 2 or 3 days at 30 °C. Individual colonies were picked and streaked on SD-His plates and incubated for 2 more days at 30 °C. One isolated colony per streak was patched on the same medium and incubated for 2 extra days at 30 °C before genotypic characterization by PCR, multiplex PCR and pulsed-field gel electrophoresis (PFGE). For PCR, total DNA was extracted from the yeast transformants as described previously [54, 54]. For PFGE, yeast total DNA was isolated in agarose plugs using the CHEF Mammalian Genomic DNA Plug kit (Bio-Rad) as previously described [27]. Yeast plugs carrying *Mccp* genomes were first treated with a cocktail of restriction enzymes (AsiSI, RsrII and FseI) and submitted to classical electrophoresis on a standard gel (1 % agarose, 1× TAE, 120 min at 120 v). After the removal of the yeast linear chromosomes, the DNA remaining in plugs were digested with BssHII and subjected to PFGE.

### Plasmid construction

Several plasmids were constructed during this study. Plasmid pMT85P*genta*-PS*lacZ*-PRS313 (Fig. S1) was built by replacing the tetracycline resistance marker driven by the spiralin promoter [PS*tet*(M) cassette] from the transposon-based plasmid pMT85PS*tet(M*)-PS*lacZ*-pRS313 [28] with the *aacA-aphD* gentamicin resistance marker (*gmR*) originating from the pMT85 minitransposon [37]. The *aacA-aphD* cassette together with its promoter was amplified by PCR using the primers Psgenta-NsiI and Psgenta-AgeI (Table S1) and the pMT85 [37] as a template. Plasmid pMT85P*genta*-PS*lacZ*-PRS313 was used to amplify the recombination template (Fig. S2) required to clone the *Mccp* genome in yeast (CReasPy-Cloning step). Replicative plasmids pMccpOric1 and pMccpOric2 were built as follows. First, a 4034 bp PstI DNA cassette carrying a tetracycline resistance gene [(*tet*(M)] was amplified using the pIVT-1 [35] as a template and the primer pair PIVT-TETF1/PIVT-TETR (Table S1) and then inserted at the PstI site of the pBluescript II KS (−) commercial plasmid (Invitrogen) to generate the pBKS-P_ivt_tetM. Second, a 1974 bp BamHI DNA cassette harbouring the *Mccp dnaA* gene surrounded by its intergenic regions (*Mccp oriC*) was PCR-amplified using *Mccp* strain 9231-Abomsa genomic DNA as a template and the primer pairs MccpOri-F/MccpOri-R. The resulting pMccpOric1 and pMccpOric2 differ from each other by the orientation of the BamHI DNA cassette (Fig. S3). The plasmid pMccpO2-CRE (Fig. S4) was derived from pMccpOric2 plasmid and was built using the NEBuilder HiFi DNA Assembly Cloning kit. Four overlapping DNA fragments were PCR-amplified (Advantage HF 2 PCR kit from Clontech) using primers described in Table S1, purified and combined at 50 °C according to the manufacturer’s instructions. DNA cassettes were designed to contain ~40 bp overlaps. The first DNA cassette corresponds to the *cre* coding sequence (1032 bp) from the pBSK_P438_Cre_Gm plasmid [46]. The second cassette corresponds to the gentamicin promoter (558pb) from the pMT85 [37]. The third cassette (4979 bp) corresponds to half of the pMccpOric2 plasmid, while the fourth one (4069 bp) corresponds to the other half of the plasmid.

gRNA plasmids constructed in this study were all derived from the parental plasmid pgRNA.AarI [25] (see section on commercial plasmids) that contains all the elements necessary for the expression of the gRNA in yeast. gRNA plasmids were constructed as described by Tsarmpopoulos *et al*. [25]: pgRNA.AarI was digested with AarI (Thermo Scientific) to remove the spacer component of the gRNA and further ligated with annealed oligonucleotide pairs. The resulting plasmids were transformed in *E. coli* and sequence-verified.

### Yeast transformation (plasmids) and yeast transformant screening

Yeast cells were transformed with ~1 µg of plasmids (pCas9 and pgRNA.AarI) using the lithium acetate protocol published elsewhere [56]. Transformants were selected by auxotrophy complementation. Yeast clones were screened by PCR for the presence of the plasmid and PCR products were sequenced. Total genomic DNA was extracted from yeast transformants as described elsewhere [54].

### *Mccp* genome transplantation into *Mcap*

Genome transplantations were performed as previously described with slight modifications [27, 28, 57]. Briefly, yeast agarose plugs containing *Mccp* genomes were digested with 3 units/plug of β-agarase I (NEB) and then transplanted in recipient cells of *M. capricolum* subsp. *capricolum* (strain California Kid^T^) devoid of restriction-modification systems (*McapΔRE* cl17.5). This latter strain, obtained by inactivation of the CCATC-restriction enzyme in the wild-type strain (ATCC 27343) [27], was grown at 30 °C in super optimal broth (SOB) [58] supplemented with 17 % (v/v) foetal bovine serum, glucose at 10 g l^−1^, 0.002 % (w/v) phenol red and penicillin at 0.5 g ml^−1^ [SOB (+) medium]. The changes to the genome transplantation protocol [28] were: (i) after the 90 min of incubation between *McapΔRE* recipient cells and *Mccp* donor genomes in the presence of 2× fusion buffer, 5 ml of m-Hayflick medium was added to the mixture; (ii) after the last centrifugation, the cell pellet was resuspended in 1 ml of m-Hayflick medium; (iii) at the end of the process, transplants were recovered on m-Hayflick solid medium supplemented with gentamicin at 300 µg ml^−1^ and incubated at 37 °C under a 5 % CO_2_ atmosphere. Isolated colonies were then picked and cultured in m-Hayflick broth supplemented with gentamicin during three passages and their respective genotypes and phenotypes were further analysed. One passage corresponds to the transfer of 10 µl of mycoplasma culture to 1 ml of medium (1/100 dilution) followed by an incubation period of ≥24 h at 37 °C.

### *Mccp* transformation

*Mccp* was transformed by a polyethylene glycol (PEG)-mediated method already described [59, 60]. Briefly, 5 ml of mid/late-log phase *Mccp* cultures grown in m-Hayflick medium were harvested at 7000 ***g*** for 15 min (12 °C), washed once with 5 ml of S/T buffer (sucrose 500 mM, Tris 10 mM, pH6.5) and resuspended in 250 µl of 0.1 M CaCl_2_. After 30 min on ice, cells (~10^9^) were mixed with 10 µg of plasmid DNA and 10 µg of yeast tRNA. Two millilitres of 70 % PEG8000 (Fisher, 10407773) were first added and let in contact for 2 min maximum, followed by 20 ml of S/T buffer. Tubes were inverted several times and centrifuged for 12 min at 12 000 ***g*** at 12 °C. The cell pellet was resuspended in 1 ml of warm m-Hayflick, incubated for 3 h at 37 °C under a 5 % CO_2_ atmosphere and directly plated undiluted and after dilution (10^−1^, 10^−2^) on m-solid Hayflick medium containing gentamicin 300 µg ml^−1^, puromycin 8 µg ml^−1^ or 0.5 µg ml^−1^ of tetracycline. Colony development was checked from the 5th day of incubation and up to 20 days (for the pMccpO2-Pgenta-CRE). Transformants were subcultured three times in 1 ml of liquid medium with selection and characterized by PCR. The PCR primer pair PIVT-TETF1/PIVT TETR (Table S1) was used to verify the presence of the tetracycline resistance marker in transformants and the PCR primer pairs Mccp-rpnA-F/Mccp-dnaN-R and Mccp-rpnA-F/AmpR-R (Table S1) were used to monitor plasmid behaviour (free versus integrated) during cell propagation.

### Molecular biology

For amplicons used in plasmid constructions, PCR was performed using the Q5 Polymerase (NEB #M0491S) following the manufacturer’s instructions. For transformant screenings (yeast and mycoplasma), PCR was performed with the Advantage2 polymerase mix (Takara #639201) following the manufacturer’s instructions. Multiplex PCR amplification was performed with the QIAGEN Multiplex PCR kit (QIAGEN #206143). The primer pairs used during simplex and multiplex PCR are all listed in Table S1.

*Mccp* agarose plugs and yeast agarose plugs were subjected to PFGE in a 1 % Bio-Rad Pulsed Field Certified Agarose (Bio-Rad #162–0137) gels in 0.5× TBE buffer (Bio-Rad #161–0733), with a CHEF-DR III PFGE system (Bio-Rad). Running conditions were set up as follows: switch angle 120 °, voltage gradient 6 V cm^−1^, ramped switch time from 50 (initial time) to 90 s (final time), duration 22 h and temperature 14 °C. After electrophoresis, the gel was stained with SYBR Gold and visualization was performed using a Vilbert Lourmat E-BOX VX2 Complete Imaging system. The Bio-Rad Marker 0.225–2.2 Mb *S*. *cerevisiae* chromosomal DNA (Bio-Rad #170–3605) was used for DNA size estimations.

### Casein digestion on milk agar

Casein digestion on milk agar was performed as previously described [61], with the following modifications: (i) milk agar plates were prepared by adding 0.8 % (w/v) dried skimmed milk powder to m-Hayflick agar medium, (ii) mycoplasmas were grown to mid-log-phase and (iii) 1 and 3 µl of mid-log-phase cultures were spotted on milk agar plates and incubated for 48 h at 37 °C under a 5 % CO_2_ atmosphere. Casein digestion was evidenced by the presence of a translucent area around the mycoplasma cells spots.

### Genome sequencing and analysis

DNA extraction was performed as described by Ruiz *et al*. [53]. Sequencing was performed by the Genome Transcriptome Facility of Bordeaux (https://pgtb.cgfb.u-bordeaux.fr/). Briefly, 100 ng of gDNA were used for the library preparation, which was performed using the QIAseq FX DNA library kit from QIAGEN. Illumina sequencing was performed on a MiSeq using V2 chemistry to produce 250 pb paired-end reads. The Illumina reads were trimmed using fastp (v.0.21.0) [62] from the 5′ end using a 4 bp sliding window with phred score <20, and reads under 30 bp were eliminated. The reads were aligned with bwa-mem (v.0.7.15) [63] taking the *Mccp* 9231-abomsa (RefSeq NZ_LM995445) as the reference genome. GATK (v.3.7) IndelRealigner [64] was then used to perform local realignment around indels, read mate coordinates were added using SAMtools (v.1.5) [65] fixmate command, duplicated reads were marked with the Picard toolkit (v.2.18.9 available at https://broadinstitute.github.io/picard), GATK HaplotypeCaller was then used with a ploidy set to 1 to call the variant positions. Changes in amino acids were identified using SnpEff (V.4.1) [66]. *De novo* assembly was also performed using the Illumina reads and Oxford Nanopore long reads to confirm chromosomal integrity and lack of recombination. Briefly, the ONT reads were filtered using filtlong (v.0.2.0, available at https://github.com/rrwick/Filtlong), and reads with a length <250 bp or sharing <87 % identity with the trimmed Illumina reads were excluded. The long reads were then assembled using canu (v1.8, available at https://github.com/marbl/canu) [67]. The initial assemblies were polished by iterative alignment of the trimmed Illumina reads as described by Talenton *et al*. [24]. The polished genomes were then compared to the *Mccp* 9231-abomsa genome sequence using Mummer4.0 (v.4.0.0.beta2 available at https://github.com/mummer4/mummer) [68] to detect potential recombinations.

### Plasmid sequencing

*Oric* plasmids were fully sequenced at Plasmidsaurus (https://www.plasmidsaurus.com/).

## Results

### Cloning of the whole *Mccp* genome in yeast

The in-yeast strategy requires three steps: (i) the cloning of the bacterial genome in yeast, (ii) the in-yeast modification of the cloned genome and (iii) the back-transplantation of the modified bacterial genome from yeast into a suitable recipient bacterium [27, 69]. Among the methods that have been described in the literature to clone a whole bacterial genome in yeast, we selected the recently developed CReasPy-Cloning method that allows one to simultaneously clone and engineer the bacterial genome in a single step [53] and, thus, to shorten the time necessary to obtain specific mutants. To do so, the yeast strain VL6-48N, carrying expression plasmids for Cas9 and a gRNA targeting the S41 serine protease encoding the gene *peptS41*, was co-transformed with whole *Mccp* genomes, as well as a repair template specific to the *peptS41* target locus on the mycoplasma genome (Fig. 1). This gene was selected because its product, the S41 serine protease, was recently identified as a putative virulence factor, while the associated phenotype (protease activity) can be easily assessed in plate cultures using a simple casein digestion assay [61].

Briefly, CReasPy-Cloning experiments were performed using two yeast clones expressing different gRNAs, both targeting *peptS41* (VL6-48N-Cas9-gRNA1 cl3 and VL6-48N-Cas9-gRNA2 cl9), and included two types of repair templates. The first template was made of (i) a centromere (CEN6), an autonomously replicating sequence (ARSH4), and the HIS3 selection marker for selection in yeast, (ii) a gentamicin resistance marker for selection in mycoplasma cells, and (iii) 60 bp recombination arms placed at each extremity, identical to the sequences flanking the target gene. The second template was indistinguishable from the first except for the presence of two 34 bp loxP sites [55] flanking the Cre targets, i.e. the gentamicin resistance marker and the yeast components that must be removed to obtain seamless mutants (Fig. S2). Following transformation, yeast transformants were analysed first by simplex PCR to confirm the presence of the *Mccp* genome and the correct deletion of the target gene, then by multiplex PCR and PFGE to assess *Mccp* genome integrity.

**Fig. 2.**
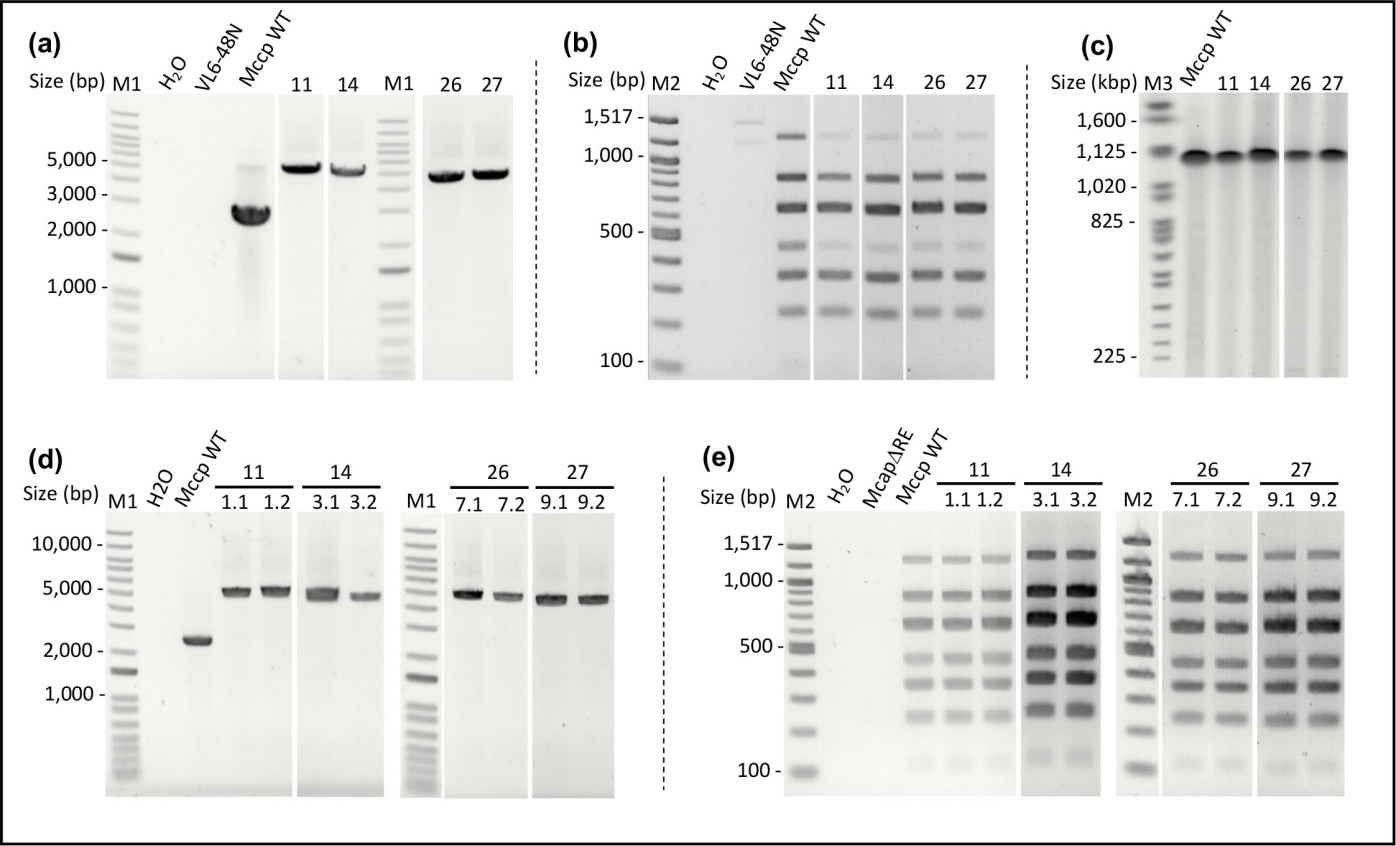
Screening of yeast transformants generated by CReasPy-Cloning (a, b, c) and further *Mccp-Ycp-ΔpeptS41* transplants (d, e). (a) Detection of the *peptS41* deletion by simplex PCR analysis with specific primers flanking the target region. The expected sizes of the PCR products before and after replacement of the target gene by the repair template are 2501 and 5044 bp (or 5112 bp for the repair template with loxP sites), respectively. A total of 40 yeast clones (20 for the repair template without loxP and 20 for the repair template with loxP sites) were screened with the primer pair Mccp-verif-PeptS41_F/Mccp-verif-PeptS41_R. Shown here are the results obtained for selected yeast clones 11 and 14 (repair template without loxP site) and 26 and 27 (repair template with loxP site). M1, 1 kb plus DNA ladder (Thermo Fisher); *Mccp* WT, wild-type *Mccp* strain Abomsa gDNA; H2O, negative control without DNA. (b) Screening for *Sc-Mccp-ycp-ΔpeptS41* and *Sc-Mccp-ycp-ΔpeptS41-lox* genome completeness by multiplex PCR. A set of seven pairs of *Mccp*-specific primers, evenly distributed around the *Mccp* genome, were designed and used in a multiplex PCR assay using 50 ng of yeast total DNA template. The reaction allows the simultaneous amplification of seven PCR fragments with sizes ranging from ~100 to 1200 bp. Yeast clones 11, 14, 26 and 27 showed the expected seven-band profile. M2, 100 bp DNA ladder (Promega); *Mccp* WT, wild-type *Mccp* strain Abomsa gDNA; H2O, negative control without DNA. (c) Validation of *Sc-Mccp-ycp-ΔpeptS41* and *Sc-Mccp-ycp-ΔpeptS41-lox* genome integrity by pulsed-field gel electrophoresis (PFGE). After removal of the yeast genomic DNA, *Sc-Mccp-ycp-ΔpeptS41* and *Sc-Mccp-ycp-ΔpeptS41-lox* genomes embedded in agarose plugs were digested with the restriction enzyme BssHII and submitted to PFGE. The single cutter BssHII should produce a single linear DNA fragment of ~1 Mbp. Similar to the *Mccp* WT positive control, yeast clones 11, 14, 26 and 27 showed a single band at a size close to 1 Mbp. M3, PFGE marker, 0.225–2.2 Mb *S. cerevisiae* chromosomal DNA (Bio-Rad); *Mccp* WT wild-type *Mccp* strain Abomsa gDNA. (d, e) Validation of the *Mccp-ycp-ΔpeptS41* and *Mccp-ycp-ΔpeptS41-lox* mutants generated by transplantation of the in-yeast modified *Mccp* genomes (cl11, 14, 26 and 27) into *McapΔRE* recipient cells by simplex PCR (d) and multiplex PCR (e). Simplex PCR was performed using the aforementioned primer pair Mccp-verif-PeptS41_F/Mccp-verif-PeptS41_R (expected band at 5112bp) and multiplex PCR were performed as in (b). All *Mccp* transplants tested (eight in total) displayed the expected profile. The images used to produce panels (a–e) are spliced from multiple gels in order to display clones of interest next to each other. Original images are available in Fig. S5.

Transformation of yeast cells with *Mccp* genomes and either of the 2 repair templates yielded 12 to >300 colonies on selective plates. Forty yeast colonies in total were picked to be analysed at the genotypic level: (i) only 10 colonies for yeast clone VL6-48N-Cas9-gRNA1 cl3, as the number of colonies present in control plates was of the same order of magnitude as in the assay plates, and (ii) 30 colonies for yeast clone VL6-48N-Cas9-gRNA2 cl9 (Table 1). Simplex PCR analysis flanking the insertion site confirmed the cloning failure for VL6-48N-Cas9-gRNA1 cl3: none of the colonies tested were found to be positive. In contrast, 13 colonies out of 30 were found to be positive for VL6-48N-Cas9-gRNA2 cl9 with amplicons of the expected sizes, either 5044 bp (repair template without loxP) or 5112 bp (repair template with loxP), while the *Mccp* wild-type (WT) control displayed a band at 2501 bp (Figs 2 and S5a). The 13 positive yeast clones were selected and their genome completeness was confirmed using an *Mccp*-specific multiplex PCR assay developed specially for this study. Eleven out of 13 yeast clones showed the expected 7 band profile (6 for the repair template without loxP and 5 for the one with loxP), suggesting that the overall genome organization was preserved in these clones (Figs 2b and S5b). To confirm these results, six samples (three per repair template) were chosen and submitted to PFGE. All yeast clones displayed an identical profile to that obtained for the *Mccp* WT positive control, presenting a single 1 Mb band as expected for BssHII cleaved *Mccp* genomes (Figs 2c and S6c). All these genotypic analyses allowed us to conclude that the *Mccp* genomes cloned in yeast were indeed devoid of *peptS41* and that their genome integrity was otherwise conserved. This validated mutant genomes were named *Sc-Mccp-ycp-ΔpeptS41* (and *Sc-Mccp-ycp-ΔpeptS41-lox* when including the loxP sites).

**Table 1.** CReasPy-Cloning experimental design and yeast transformation results

Tube no.	Yeast clones	Repair template (300 ng)	*Mccp* WT genomes (vol.)	No. of yeast colonies on selective plates (SD-His)	No. of colonies picked	No. of positive clones (simplex PCR)
1	VL6-48N-Cas9-ARNg1 cl3	–	–	0	–	
2		RT-*Mccp-peptS41*	–	19	–	
3		RT-*Mccp-peptS41-loxP*	–	48	–	
4		RT-*Mccp-peptS41*	20 µl	12	5*	0/5
5		RT-*Mccp-peptS41-loxP*	20 µl	34	5*	0/5
6	VL6-48N-Cas9-ARNg2 cl9	–	–	0	–	
7		RT-*Mccp-peptS41*	–	37	–	
8		RT-*Mccp-peptS41-loxP*	–	120	–	
9		RT-*Mccp-peptS41*	20 µl	>300	15^†^	8/15
10		RT-*Mccp-peptS41-loxP*	20 µl	>300	15^†^	5/15
**TOTAL**					40	13/40

* Ten colonies were picked for yeast clone VL6-48N-Cas9-gRNA1 cl3 (five per repair template), as the number of colonies present in control plates were of the same order of magnitude as in the ‘assay’ plates (lanes 2/3 vs 4/5), indicating a probable cloning failure.

† Thirty colonies were picked for yeast clone VL6-48N-Cas9-gRNA2 cl9 (15 per repair template), as the number of colonies present in control plates was 3 to 10 times lower than in the ‘assay’ plates (lanes 7/8 vs 9/10).

### Back-transplantation of *Mccp* genomes and mutant characterization

The next step in the in-yeast engineering process is the transplantation of the edited genome into a suitable recipient bacterium to generate cognate mutants (Fig. 1). For this purpose, intact *Sc-Mccp-ycp-ΔpeptS41* and *Sc-Mccp-ycp-ΔpeptS41-lox* genomes, each isolated from two different yeast clones, were back-transplanted into recipient cells of *Mcap∆RE*, a *M. capricolum* subsp. *capricolum* mutant free of restriction enzyme-coding genes [27].

Initial attempts to transplant *Mccp* genomes into *Mcap∆RE* failed. The genome transplantation protocol was thus modified to best fit *Mccp* needs: the SP5 medium used for fusion buffer dilution, cell regeneration, and transplant selection was replaced with m-Hayflick medium and the gentamicin concentration used for transplant selection in m-Hayflick plates was reassessed to 300 µg µl^−1^.

Thus revised, the protocol allowed us to rescue colonies on the transplantation assay plates, while there was no colony on either the no-recipient-cell control or the mock-transplanted control plates. Fifteen to 36 putative transplants per yeast clone appeared on selective plates containing gentamicin. Twenty-four colonies total were picked (six per yeast clone) and analysed both by simplex PCR (Figs 2d and S5d) and multiplex PCR (Figs 2e and S5e). All were identified as *Mccp* lacking the target gene *peptS41*. These clones were named *Mccp-ycp-ΔpeptS41* (and *Mccp-ycp-ΔpeptS41-lox* when including the loxP sites).

**Fig. 3.**
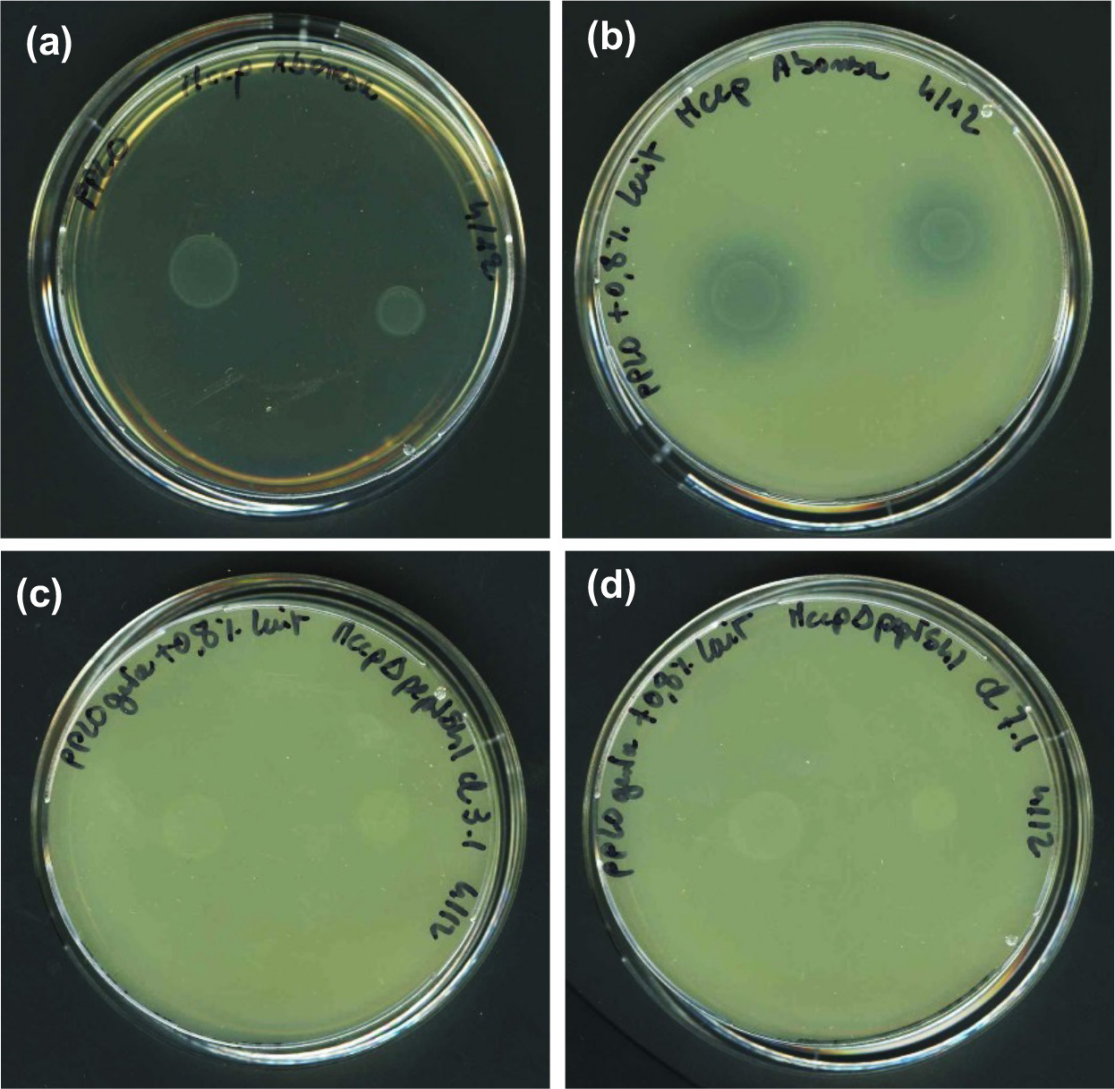
Functional analysis of *Mccp-ycp-ΔpeptS41* and *Mccp-ycp-ΔpeptS41-lox* mutants. Casein digestion was assessed on modified Hayflick’s agar plates supplemented with 0.8 % (w/v) milk. (a) *Mccp WT* on a standard agar plate; (b) *Mccp* WT on a milk agar plate; (c, d) *Mccp-ycp-ΔpeptS41* mutant cl 3.1 (c) and *Mccp-ycp-ΔpeptS41-lox* mutant cl7.1 (d) on milk agar plates. A translucent area of casein digestion was observed around *Mccp* WT colonies, indicating functional S41 serine protease secretion and activity (b) . In contrast, no halo was visible around the colonies of *Mccp* mutants cl3.1 (c) and cl7.1 (d) . For both *Mccp* WT and mutants, two volumes of culture were spotted on the plates (1 and 3 µl) .

Among the clones with the expected profile at the genotypic level, two were randomly selected to be characterized at the phenotypic level: *Mccp-ycp-ΔpeptS41* cl3.1 and *Mccp-ycp-ΔpeptS41-lox* cl7.1. Both clones, as well as the *Mccp* WT strain, were grown in modified m-Hayflick broth and further seeded on m-Hayflick agar plates supplemented with milk to assess the ability of the mycoplasma cells to digest casein. The results are shown in Fig. 3 *. Mccp* WT, which secretes the serine protease PeptS41, was able to digest casein, as evidenced by the presence of a translucent halo around the colonies (Fig. 3b). On the other hand, the two selected mutants, which presented unchanged growth characteristics as compared to the WT, were unable to digest casein, as indicated by the absence of translucent halo around the colonies (Fig. 3c, d). These results clearly indicate that the S41 serine protease encoded by *peptS41* was fully inactivated in both mutants using the in-yeast engineering approach. To our knowledge, the *Mccp peptS41* knockouts produced here constitute the first site-specific mutants reported for this species.

### Development of a functional *oriC* plasmid for heterologous protein expression in *Mccp*

Following in-yeast engineering, yeast genetic elements and an antibiotic resistance marker remain in the genomes of selected mutants, which may be detrimental for downstream applications such as animal studies. To allow their removal, we developed a dedicated tool based on the Cre–lox system, which relies on a site-specific Cre recombinase and its loxP recognition site. While loxP sites were successfully introduced into some *Mccp* mutants during in-yeast engineering (i.e*. Mccp-ycp-ΔpeptS41-lox* cl7.1), systems allowing the transient expression of Cre in *Mccp* were lacking.

Since *oriC* replicative plasmids have proven useful for the expression of heterologous proteins in closely related *Mycoplasma* species [16, 19, 20], we decided to produce an *oriC* plasmid for use in *Mccp*. Artificial *oriC* plasmids from *Mycoplasma* spp. have a fairly similar architecture [17, 18, 70, 71]; they are generally based on the pBS(+) plasmid (Stratagene) and contain the chromosomal *dnaA* gene and surrounding DnaA box sequences, allowing replication in the target species, and an antibiotic resistance cassette. However, no antibiotic resistance cassette was described in the literature for use in *Mccp*, while few data were available regarding the natural resistance of *Mccp* to various antibiotics. Thus, following the determination of the *Mccp* MIC for puromycin and tetracycline in m-Hayflick medium (which were 0.8 and 0.03 µg ml^−1^ respectively), we tested a battery of puromycin and tetracycline resistance cassettes present in available minitransposons (Fig. S6). More specifically, *Mccp* was transformed with MiniPS*puro* [72]*,* MiniP438*puro*, MiniPS*tet(M*) [72], pIVT-1 [35] and pMT85PS*tet(M)-*PS*lacZ*-pRS313 [28] using a 70 % PEG-based protocol with either 8 µg ml^−1^ of puromycin or 0.5 µg ml^−1^ of tetracycline, as required for selection.

Concerning puromycin selection, although colonies were observed for the two plasmids tested, they were also present in control plates, meaning that spontaneous mutants emerged in the selective medium. Concerning tetracycline selection, colonies were observed for pIVT-1, while none and few (10 times fewer) were observed for MiniPS*tet(M*) and pMT85PS*tet(M)-*PS*lacZ*-pRS313, respectively (Table S2). As expected, all PCR-tested tetracycline resistant colonies from pIVT-1 carried the tetracycline cassette, while none of the puromycin-resistant colonies carried the puromycin cassette.

Based on these results, two *Mccp oriC* plasmids with the tetracycline resistance cassette originating from the pIVT-1 plasmid were built: pMccpO1 and pMccpO2, which differed from each other in the orientation of the 2 kb *oriC* region (Fig. S3). Both plasmids were transferred into *Mccp* cells along with the pIVT-1. The experiment was performed in triplicate (Table 2). Between ~4100 and ~7700 colonies appeared on selective plates in pMccpO2 transformations versus only 0 to 2 colonies when using pMccpO1. Concerning the positive control pIVT-1, the results obtained were very similar to those obtained previously, with a mean transformation efficiency of ~2.64×10^−9^ transformants c.f.u./total c.f.u. µg^−1^ of plasmids over three experiments. Ten days after transformation, three colonies were picked from pMccpO2 plates after the three assays, subcultured during three passages in m-Hayflick medium with tetracycline and checked by PCR using the PIVT-TETF1 and PIVT-TETR primers (Table S1). All nine samples presented the expected 4030 bp *tet(M*) amplicon (Fig. S7, red column), indicating that all contained the plasmid. The state of the pMccO2 plasmid, either in free form or integrated in the chromosome by homologous recombination at the *dnaA* gene, was then assessed. The plasmid was found totally free in four of the nine clones analysed, and in mixture in the other five. The finding of extrachromosomal DNA molecules in *Mccp* transformants demonstrated that pMccpO2 is a replicative plasmid. The state of integration of pMccO2 was also evaluated at a later passage, after growth in presence or absence of antibiotics (Fig. S7, blue and green columns). The data showed that pMccpO2 can be maintained free, as extra-chromosomal molecules, in some clones up to at least six passages after transformation in the presence of antibiotics, and that it can, by contrast, be rapidly lost in an antibiotic-free environment. We concluded from these experiments that we could use the pMccpO2 plasmid to express the heterologous *cre* gene over a few generations, and that it would then be possible to get rid of the pMccpO2-CRE plasmid simply by removing the selection pressure.

**Table 2.** Transformation of *Mccp* with *Mccp oriC* plasmids

	Plasmid (10 µg)	No. of colonies on selective plates†	Transformation efficiencies‡	No. of colonies picked
Assay 1*	1- pMccpO1	nd: 0	–	–
	2- pMccpO2	10^−2^: 41	7.6×10^−8^	3
	3- pIVT-1	nd: 54	1×10^−9^	–
	4 - No DNA (tet0.5)	nd: 0	–	–
Assay 2*	5- pMccpO1	nd: 0	–	–
	6- pMccpO2	10^−2^: 66	1.58×10^−7^	3
	7- pIVT-1	nd: 56	1.34×10^−9^	–
	8-No DNA (tet0.5)	nd: 0	–	–
Assay 3*	9- pMccpO1	nd: 2	0.44×10^−10^	–
	10- pMccpO2	10^−2^: 77	1.71×10^−7^	3
	11- pIVT-1	nd: 252	5.6×10^−9^	–
	12- No DNA (tet0.5)	nd: 0	–	–

*The experiment was performed in triplicate.

†nd, transformed cells were spread on m-Hayflick selective plates directly after transformation. 10^−2^, transformed cells were diluted 100-fold before spreading on m-Hayflick selective plates.

‡Number tfs c.f.u./total c.f.u. µg^−1^ of plasmids.

At this stage of the experiment, we had not only constructed a functional *oriC* plasmid for *Mccp*, but we had also obtained a highly efficient plasmid, with transformation efficiencies of ~7.6×10^−8^ to ~1.71×10^−7^ transformant c.f.u./total c.f.u. µg^−1^ of plasmid DNA (i.e. 30 to 100 times higher than for plasmid pIVT-1).

### Removal of unwanted genetic sequences in *Mccp* mutants using pMccpO2-CRE

**Fig. 4.**
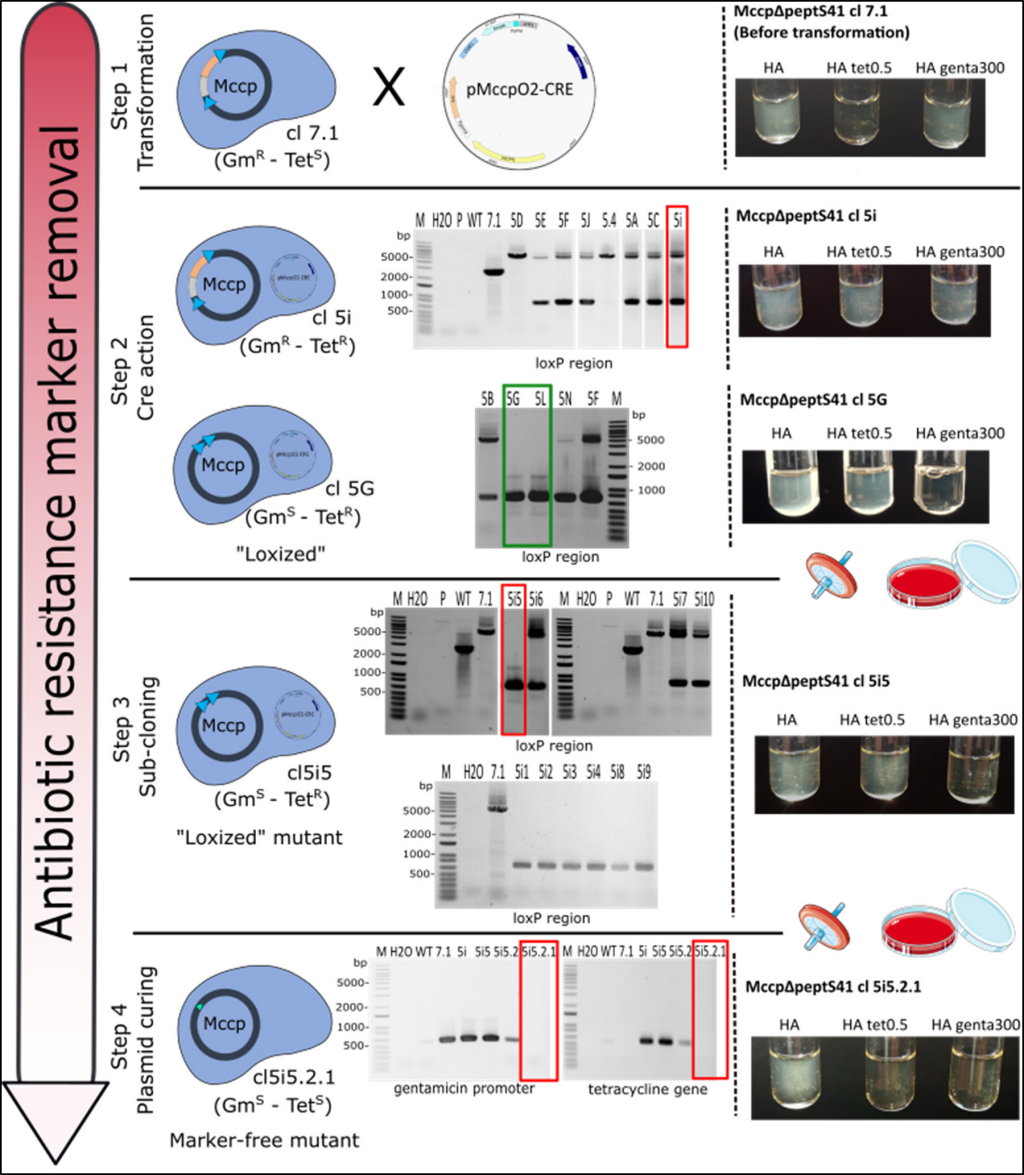
Isolation of marker-free *Mccp* mutants through the action of the Cre recombinase. Step 1: *Mccp-ycp-ΔpeptS41* mutant cl7.1, which carried the gentamicin resistance marker/yeast elements cassette surrounded by loxP sites (loxP region) in place of the S41 serine protease-encoding gene, was transformed with the pMccpO2-CRE plasmid carrying a tetracycline resistance marker, as well as the *cre* encoding gene under the control of the gentamicin promoter. Before transformation, *Mccp-ycp-ΔpeptS41* mutant cl7.1 was able to grow in m-Hayflick medium (HA) with 300 µg ml^−1^ gentamicin, as shown in the picture. Step 2: after transformation, *Mccp* clones selected on m-Hayflick solid agar with 0.5 µg ml^−1^ tetracycline were picked in 1 ml of liquid m-Hayflick medium containing the same concentration of tetracycline and further propagated in 1 ml of the same medium without antibiotic. PCR analysis were performed using primers Verif-MccpPeptS41-F/R flanking the loxP region. In parallel, growth assays were carried out in m-Hayflick medium, m-Hayflick medium supplemented with tetracycline and m-Hayflick medium with gentamicin, as illustrated for cl5i and cl5G. Step 3: clone 5i was filter-cloned (0.22 µm) and plated on m-Hayflick solid agar. Ten subclones were picked and sub-cultured in 1 ml of m-Hayflick medium, and analysed by PCR using the same primer pairs used in step 2. Growth assays were also performed as shown for cl5i5. Step 4: *MccpΔpeptS41* subclone 5i5 was filter-cloned and plated in m-Hayflick medium (this step was repeated twice) and finally checked by PCR using primers pairs PgentaF/R and Tet1/Tet2, specific for the gentamicin promoter and the tetracycline resistance marker, respectively. Growth was monitored as in step 2 and 3. M, 1 Kb plus DNA ladder (Thermo Fisher); H2O, negative control without DNA; P, plasmid pMccpO2-PgentaCRE; WT, wild-type *Mccp* strain Abomsa gDNA; 7.1 mutant *Mccp-ycp-ΔpeptS41* cl7.1; HA, m-Hayflick medium. The images used to produce Fig. 4 are spliced from multiple gels in order to display some clones of interest. Original images are available in Fig. S9.

In order to produce unmarked mutants cleared from all DNA sequences added during the genome engineering process (Fig. 1), we constructed the pMccpO2-CRE plasmid in which the *cre* gene was placed under the control of the gentamicin promoter (Fig. S4) and used it to transform the *Mccp-ycp-ΔpeptS41-lox* cl7.1 mutant (Fig. 4, step 1). Colonies appeared on m-Hayflick tetracycline plates up to 20 days after transformation (Table 3). A total of 29 colonies were picked and cultured in m-Hayflick liquid medium with tetracycline at different time points (12 at day 10; 2 at day 15, and 15 at day 20) and they were all incubated until observation of turbidity. Unexpectedly, only one (cl5.4) of the samples taken at days 10 and 15 showed signs of growth. In contrast, 11 samples out of 15 collected at day 20 showed growth.

**Table 3.** Transformation of *Mccp* with *oriC* plasmid pMccpO2-CRE

	Plasmid (10 µg)	No. of colonies on selective plates†	Transformation efficiencies‡	No. of colonies picked at day 10 after transformation	No. of colonies picked at day 15 after transformation	No. of colonies picked at day 20 after transformation
***Mccp* ∆peptS41 cl7.1**	pMccpO2*	10^−1^: 245	1.38×10^−7^	–	–	–
	pMccpO2-CRE	10^−1^: 48	<0.26×10^−7§^	12 (cl. 5.1 to 5.12)	2 (cl. 5.13 to 5.14)	15 (cl. 5A to 5O)
	No DNA	nd: 0	–			

*pMccpO2 was used as a positive control of transformation.

†nd, transformed cells were spread on m-Hayflick selective plates directly after transformation. 10^-1^, transformed cells were diluted 10-fold before spreading on m-Hayflick selective plates.

‡Number tfs c.f.u./total c.f.u. µg^−1^ of plasmids.

§Transformation efficiency was probably overestimated as some (mostly early-picked) colonies did not grow in a liquid selective medium (PPLO tet0. 5 µg µl^−1^).

All positive cultures (12 in total) were passaged once in m-Hayflick broth without tetracycline and then analysed by PCR using the primer pair Verif-MccpPeptS41-F/R that flanks the loxP region overlapping the gentamicin resistance marker and all yeast elements targeted by the Cre (Fig. S8). We chose to remove the selection pressure immediately after the first passage in broth to avoid a possible integration of the pMccpO2-CRE plasmid at the *oriC* chromosomal locus. PCR profiles revealed that (i) 2 clones (cl. 5G and 5L) out of 12 presented the 1-band profile at ~720 bp expected following Cre recombinase activity (accordingly, these clones lost their capacity to grow in m-Hayflick with gentamicin) and (ii) 10 clones had a 2-band mixed profile with one band at ~720 and another at ~5112 bp, identical to the parental strain *Mccp-ycp-ΔpeptS41-lox* cl7.1 (Fig. 4, step 2). These latter clones were able to grow in m-Hayflick medium supplemented with either tetracycline or gentamicin (as exemplified by cl5.i). Since such a pattern was most likely due to the presence of a mixed population of mycoplasma cells within a same ‘clone’ (mixture of cells with excised and non-excised loxP cassettes), one of these clones was selected for subcloning experiments. *Mccp-ycp-ΔpeptS41-lox* clone 5i was filter-cloned (0.22 µm) and plated in m-Hayflick agar without antibiotic. Ten subclones were picked in m-Hayflick broth and analysed by PCR using the same pair of primers. Seven clones out of 10 showed a unique ~720 bp band, suggesting that the loxP cassette was properly removed from the corresponding *Mccp* genomes. This result was confirmed by the absence of growth in m-Hayflick medium supplemented with gentamicin (as shown for *Mccp-ΔpeptS41* cl5i5), while growth was clearly visible in non-selective m-Hayflick medium (Fig. 4, step 3).

Surprisingly, growth was still visible in m-Hayflick medium with tetracycline, suggesting that replicative pMccpO2-CRE was still present in some cells despite the lack of selection pressure. This was also the case for cl 5G and 5L (Fig. 4, step 2). In order to obtain a mutant free of any marker, *Mccp-ΔpeptS41* subclone 5i5 was filter-cloned and plated in m-Hayflick medium (step repeated twice) and then checked by PCR using two pairs of primers (PgentaF/R and Tet1/Tet2), specific for the gentamicin and tetracycline resistance markers, respectively (Table S1). No amplicon was obtained for either gene for the subclone 5i5.2.1. In contrast, its parental lineage (cl7.1 and subclones 5i, 5i5 and 5i5.2) displayed the expected amplicons at 600 bp (gentamicin promoter) and 535 bp (tetracycline gene). In accordance with these results, growth of subclone 5i5.2.1 was only obtained in non-selective m-Hayflick medium (Fig. 4, step 4). Hence, after two steps of subcloning, we were able to isolate a *Mccp* mutant lacking the S41 serine protease-encoding gene and cleared of all unwanted sequences (*Mccp-ΔpeptS41* subclone 5i5.2.1). This whole experiment was repeated once in exactly the same conditions. At step 2, we selected cl2.2, which harboured a mixed profile (as previously observed for cl5i), and following one round of filtration and plating, we recovered the gentamicin- and tetracycline-sensitive *Mccp-ΔpeptS41* subclone 2.2.7.

These results indicate that seamless targeted mutants can be produced in *Mccp* when in-yeast methods are combined with in-mycoplasma tools, and more particularly, with *OriC* plasmids harbouring the Cre–lox recombination system.

### Evaluation of *Mccp* genome stability during the engineering process by whole-genome sequencing

The production of unmarked *Mccp-ΔpeptS41* mutants required several successive steps. First, *Mccp-ycp-ΔpeptS41* mutants in which the *peptS41* gene was replaced by a cassette carrying some yeast elements and an antibiotic resistance marker had to be produced. Second, newly generated *Mccp-ycp-ΔpeptS41* mutants had to be transformed with the Cre-expressing plasmid pMccpO2-CRE to remove all undesired sequences. Third, *Mccp-ΔpeptS41* mutants had to be propagated over several generations in a medium without selection pressure in order to lose the pMccpO2-CRE plasmid that was replicating in the mycoplasma cells.

Hence, for this first major genome engineering work on *Mccp*, we found it important to verify that the mutants generated at the end of the work carried the designed modifications and did not acquire significant mutations during *in vitro* passages that may compromise other cell functions. For this purpose, subclones *Mccp-ΔpeptS41* 5i5.2.1 and 2.2.7 were selected along with the parental *Mccp* Abomsa WT strain for whole-genome sequencing and comparison to the *Mccp* 9231-Abomsa reference genome (RefSeq NZ_LM995445).

Not surprisingly, the sequence of the *Mccp* Abomsa WT strain was almost identical to the reference sequence previously generated from this same strain, with only four variant positions observed across the entire genome, all in intergenic regions (Table S3). Three of those variant positions were conserved in all subsequent strains, while the one at position 1 015 280 was different in the WT strain and the subclones. This position is at the beginning of a stretch of 19 adenines in the reference sequence, while 20 and 18 adenines were identified in our WT strain and in the subclones, respectively (the last 2 lines of Table S3). This position apart, the *Mccp-ΔpeptS41* cl 5i5.2.1 and 2.2.7 subclones showed a very low number of mutations compared to the WT strain, giving a total of seven and four accumulated mutations, respectively, across their genomes, excluding the targeted deletion. All mutations observed were in intergenic regions, with the exception of three in clone 5i5.2.1, which occurred in coding sequences, as well as a mutation in one of the two 16S rRNA operons observed in clone 2.2.7 (Table S3). Polymorphisms in the two *Mccp* rrn operons has already been described and are known to occur in nature [49, 73, 74]. Those mutations did not prevent genome transplantation and the resulting transplants did not show noticeable growth defects.

## Discussion

In this study, we have developed a series of key genetic tools and techniques that allow the modification of *Mccp* genomes with ease to produce mutants of interest. The work presented here was performed with *Mccp* strain 9231-Abomsa. However, since *Mccp* is a monomorphic mycoplasma with extremely low levels of intraspecific genetic diversity [75, 76], these tools will most likely be suitable for all strains of this taxon. A first proof of this has already been obtained in our laboratory. The in-yeast genome engineering process was indeed recently applied to two other *Mccp* strains from Niger and Tanzania, and the corresponding mutants were obtained with the same efficacy as presented here [77].

The first genetic tool developed in this work for use in *Mccp* is the whole-genome engineering platform based on the yeast *S. cerevisae*: the *Mccp* genome was simultaneously cloned and engineered in yeast using CReasPy-Cloning and then transplanted into *Mcap∆RE* recipient cells, resulting in the production of the first *Mccp* site-specific mutants. Although the *Mccp* genome was cloned in yeast without difficulty, one of the two initially selected yeast clones expressing different gRNAs did not allow the cloning of the *Mccp* genome. This is not unusual when attempting to clone or edit bacterial genomes in yeast using the CRISPR/Cas9 editing system and may be explained by the fact that the gRNAs cloned into pgRNA plasmids to specifically target a selected locus are not always as effective as predicted [77]. In both the aforementioned and current study, gRNA selection was performed using the CRISPR Guide RNA design tool implemented in the cloud-based platform Benchling (https://www.benchling.com/). This tool allows the visualization of all putative gRNAs in a given sequence and provides ‘on-target’ [78] and ‘off-target’ [79] scores for each of them, for straightforward comparison and selection of higher activity and lower off-target effects. While most of the predictions are accurate, they do not always correlate with experimental results.

To complete the engineering process, the cloned/engineered *Mccp* genome must be transplanted into appropriate recipient cells. Labroussaa *et al*. showed that the highest transplantation efficiencies are obtained when using donor genomes isolated from species most closely related to the recipient [28]. Since *Mccp* is extremely closely related to *Mcap* (they belong to the same species and share 99.78 % similarity at the core proteome level; Sirand-Pugnet, personal communication), we decided to apply, without modification, the protocol developed in 2009 to transplant the *Mycoplasma mycoides* susbp. *capri* donor genome into *McapΔRE* recipient cells [27], and reproduced in 2016 by Labroussaa *et al*. [28]. Unexpectedly, the first experiments failed and modifications to the initial protocol concerning growth medium and antibiotic concentration were required in order to obtain the first *Mccp* transplants further identified as *Mccp-ycp-ΔpeptS41* mutants.

In-yeast engineering is a very powerful and flexible technique, offering the opportunity to produce all kinds of modifications in bacterial genomes cloned in yeast, which can go as far as the production of entirely synthetic genomes [69, 80]. However, these methods present the major drawback of requiring the introduction of foreign sequences into the genomes of interest, namely a yeast origin of replication, a centromere and markers for auxotrophy and antibiotic resistance. Upon mutant isolation, these genetic elements remain in the modified genomes. This may be of no significance for many subsequent applications, but the presence of these components is clearly detrimental to the implementation of other ones, such as *in vivo* host–pathogen interaction studies or vaccine strain development. These reasons lead us to demonstrate that it was possible to produce *Mccp* mutants free of unwanted sequences. In order to reach this goal, it was necessary to deliver a temporary system capable of cutting and stitching the genome at specific loci. We chose to work with the Cre–lox site-specific recombination system, which was shown to be functional in other mycoplasma species [39, 46, 47, 81], and to temporarily express it into the cells using an *oriC* plasmid, since replicative plasmids have proven very useful for the expression of heterologous proteins in closely related *Mycoplasma* species [16, 19, 20]. After having determined that the tetracycline resistance cassette of *Tn916* carried by pIVT-1 [35] was more efficient for selection in *Mccp* than those carried by MiniPS*tet*(*M*) [72] and pMT85PS*tet*(*M*)*-*PS*lacZ-*pRS313 [28], we used it to build two *Mccp oriC* plasmids. Unexpectedly, only one of the two constructs yielded transformants. Whole-plasmid sequencing indicated that they were identical except for (i) the orientation of the *Mccp oriC* (as designed) and (ii) one base at one extremity of the ColE1 sequence (an additional A in a polyA homopolymer). Although essential for plasmid replication in *E. coli*, the ColE1 sequence has no impact on the *oriC* plasmid behaviour in *Mccp* cells. We thus concluded that the orientation of the *oriC* cassette in the plasmid influenced the plasmid replication, a fact previously reported in *Mycoplasma feriruminatoris* (Labroussaa, personal communication). In our case, it appears that when the *oriC* is oriented in the same direction as the tetracycline resistance cassette, the plasmid can no longer replicate in *Mccp* cells.

The successful *Mccp oriC* plasmid pMccpO2 allowed the recovery of a large number of transformants and was used to induce the expression of the Cre recombinase in the *Mccp-ycp-ΔpeptS41* mutants in order to remove unwanted marks. Colonies appeared 10 days or more after transformation. Unexpectedly, only colonies that appeared last were able to grow in tetracycline selective broth. PCR screening showed that most often those colonies did not represent individual clones (e.g*.* cl5i and cl2.2, Fig. 4), and that it was necessary to go through at least one filter-cloning step to isolate pure clones that had (i) excised the loxP cassette (ARSH4-CEN3-HIS3-P_genta_-gentamicin) from their genome and (ii) eliminated the replicative plasmid pMccpO2-CRE carrying the Cre recombinase encoding gene and, most importantly, the tetracycline resistance marker. The number of subcultures and cloning steps must be limited as much as possible so as to minimize the chances of plasmid integration into the genome. Indeed, it has been shown here in *Mccp* (Fig. S7), but also in several other mycoplasma species, that *oriC* plasmids can integrate the genome by homologous recombination via a crossover at the chromosomal replication origin [17, 70, 82]. Passaging and, most importantly, cloning steps that can fix spontaneous mutation events arising during *in vitro* culture, must also be kept to a minimum in order to avoid any genetic drift in the selected mutants that may alter off-target features and lead to attenuation. In our work, we were able to recover many marker-free *Mccp-ΔpeptS41* clones after a few passages in non-selective broth and one (cl 2.2.7) to three filter-cloning steps (cl5i5.2.1).

Whole-genome sequence analysis of two of these clones (cl 5i5.2.1 and cl 2.2.7) showed an extremely low number of mutations between the modified strains and their parental counterpart. This was particularly true for cl 2.2.7, which had been selected after a single round of filter-cloning following Cre recombinase treatment. These results show that the implementation of the three-step procedure developed here for the production of marker-free, site-specific *Mccp* mutants (Fig. 1) is reliable and not mutagenic, and can thus be used with confidence. Each of these steps does not require the analysis of a large number of clones to find the one(s) of interest. The most delicate step is certainly the last one, which consists in obtaining a clone of interest without any trace or marker, as it requires one or more filtration/sub-cloning steps to isolate a pure clone.

Adaptation of the in-yeast genome engineering process to *Mccp* opens up new perspectives in terms of genome editing of this important pathogen. Large-scale modifications of the genome can be envisaged, such as those carried out in the *M. feriruminatoris* and *M. mycoides* subsp. *capri* genomes, in which 6 and 10 % of the original genome, respectively, were deleted in order to produce attenuated strains [22, 24]. This is actually crucial for the complete inactivation of virulence traits that are encoded by multiple gene copies in mycoplasma genomes, including *Mccp*, as is the case for polysaccharide biosynthesis [22, 83]. Generation of such site-specific mutants free of undesired marks will be instrumental in the identification of *Mccp* genes involved in pathogenesis by enabling host–pathogen interaction studies for the functional analysis of this mycoplasma both *in vitro* and *in vivo*. Furthermore, this new toolbox for *Mccp* genome engineering offers countless perspectives for the rational design of improved vaccinal strains, whether for use as live or as killed vaccines, by allowing the deletion and integration of genetic elements to obtain desired features, such as attenuation, improved growth, thermostability and genetic stability, as well as immunogenicity, modulation of the host’s immune response, differentiation of vaccinated from infected animals, or even the expression of heterologous antigens [24], which may pave the way for the development of multivalent vaccines.

This work started with the assumption that *Mccp* was a fastidious and genetically intractable organism. It ends with a very different view, as the *Mccp* genetic toolbox is now well filled and contains the complete process for genome engineering in yeast, a replicative plasmid for gene cloning and expression, transposon-based plasmids for gene inactivation or gene expression, two antibiotic resistance markers for mutant selection and the Cre–lox system for removal of undesired marks. This will not only be instrumental in the functional genomics approach to the identification of *Mccp* virulence traits for a better understanding of CCPP pathogenicity, but should also allow the production of rationally designed, improved vaccines for the control of this devastating disease.

## Supplementary Data

Supplementary material 1

Supplementary material 2

Supplementary material 3
