# Supplementary material for: A toolbox for manipulating the genome of the major goat pathogen, Mycoplasma capricolum subsp. capripneumoniae
Source: Microbiology (Reading). 2024 Jan 9;170(1):001423. doi: 10.1099/mic.0.001423 (PMC10866025; doi:10.1099/mic.0.001423)
Supplement: Supplementary material 1 [file mic-170-1423-s001.pdf]

Supplementary material

A toolbox for manipulating the genome of the major goat pathogen, *Mycoplasma capricolum* subsp. *capripneumoniae*.

Authors' names

Géraldine Gourgues[1], Lucía Manso-Silván[2,3], Catherine Chamberland[4], Pascal Sirand-Pugnet[1], François Thiaucourt[2,3], Alain Blanchard[1], Vincent Baby[5], Carole Lartigue[1§]

Affiliations

[1]Univ. Bordeaux, INRAE, BFP, UMR 1332, F-33140 Villenave d'Ornon, France

[2]CIRAD, UMR ASTRE, F-34398 Montpellier, France.

[3]ASTRE, Univ Montpellier, CIRAD, INRAE, F-34398, Montpellier, France.

[4]Université de Sherbrooke, département de biologie, Sherbrooke, J1K 2R1, Canada

[5]Université de Montréal, Faculté de médecine vétérinaire, Saint-Hyacinthe, Québec, J2S 2M2, Canada

[§] **Corresponding author and email address**

Carole Lartigue

Address: INRAE, Equipe Mollicutes, UMR 1332 BFP, Bâtiment IBVM - A4, 71, Avenue Edouard Bourlaux, CS 20032, F-33883 VILLENAVE D'ORNON CEDEX, France

Phone: + 33 5 57 12 23 59

Email: carole.lartigue-prat@inrae.fr

This file includes

Supplementary Figures S1 to S9

Supplementary Table S2

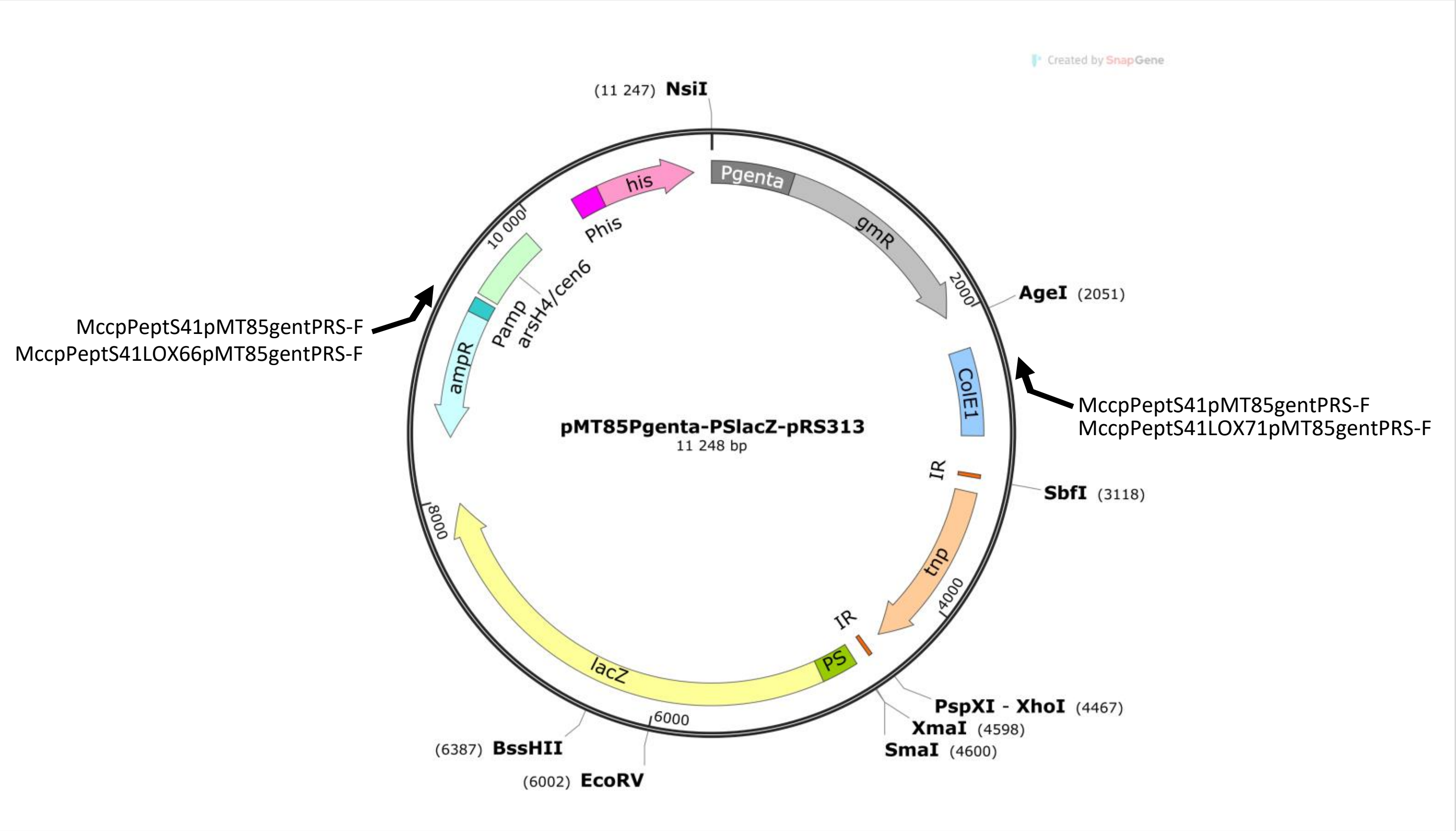

Figure S1. Map of the plasmid pMT85Pgenta-PSlacZ-pRS313. This plasmid derives from the transposon based-plasmid pMT85PS*tet(M)*-PS*lacZ*-pRS313[1]. It harbors the *aacA-aphD* gentamicin resistance gene (g*mR*) and a transposase encoding gene (*tnp*) flanked by two inverted repeats (IR) from the Tn*4001* transposon. This plasmid also contains (i) the ColE1 origin and an ampicillin resistance gene (*ampR*) for propagation and selection in *Escherichia coli*, (ii) an autonomous replicating sequence (ARSH4), a centromere (CEN6) and a histidine marker (HIS3) for propagation and selection in the yeast *Saccharomyces cerevisiae* and (iii) the beta-galactosidase encoding gene (*lacZ*) under the spiralin promoter (PS) that can be used to screen some recombinant mycoplasma species.

Recombination templates required for the CReasPy-cloning method were amplified from the pMT85P*genta*-PS*lacZ*-pRS313 plasmid using the primer pairs MccpPeptS41pMT85gentPRS-F / MccpPeptS41pMT85gentPRS-F (4,481bp) and primer pairs MccpPeptS41LOX66pMT85gentPRS-F / MccpPeptS41LOX71pMT85gentPRS-F (4,549bp). Primers were composed of 20-bases identical to the plasmid sequence and ~60-bases floating tails identical to the sequences surrounding the *Mccp* target gene MCCP001_RS01320. Primers pair MccpPeptS41LOX66pMT85gentPRS-F / MccpPeptS41LOX71pMT85gentPRS-F also includes a 34-base-long lox site (**Table S1**). The plasmid map was created using SnapGene.

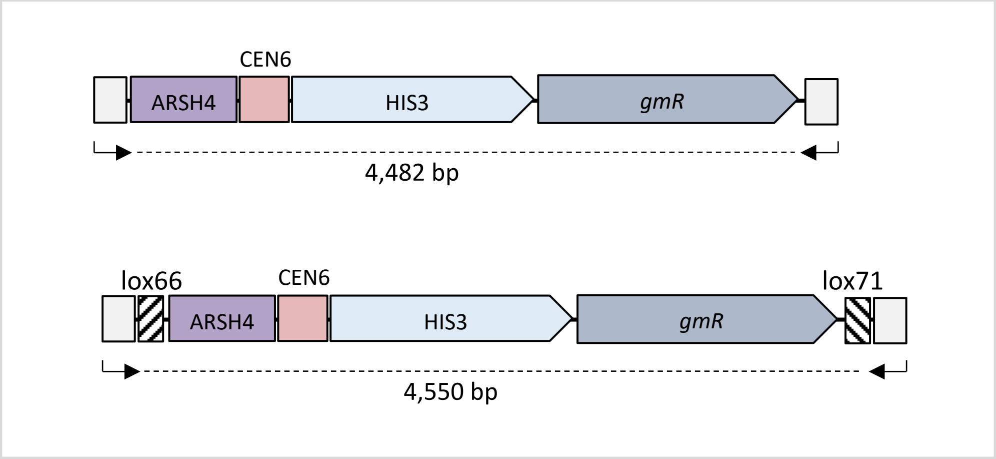

Figure S2: Schematic representation of the recombination templates used during the CReasPy-cloning experiment. Two recombination templates were used in this study. Both carry the yeast elements (ARSH4, CEN6, HIS3), the gentamicin resistance marker (*gmR*) and, at each extremity, 60pb-hooks with sequences identical to those flanking the target gene (light grey boxes). The recombination template at the bottom also includes two loxP sites (lox66 and lox71)[2] so that DNA sequences added during the genome engineering cycle can be removed if required. The size of the recombination templates is indicated.

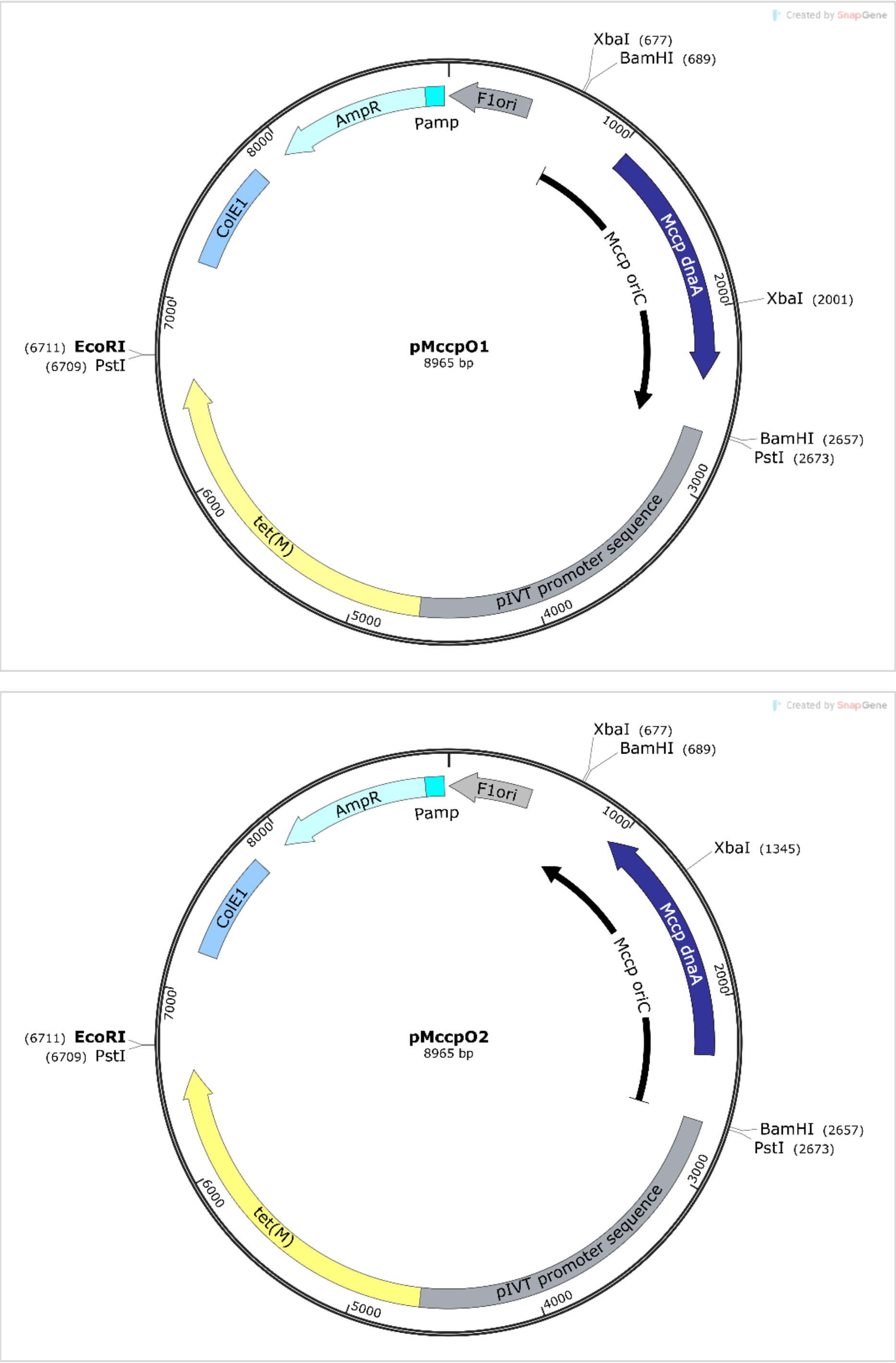

Figure S3. Maps of the *OriC* replicative plasmids pMccpO1 and pMccpO2. These plasmids derived from the pBluescript II KS (-) commercial plasmid (2,961bp), which harbors the ColE1 origin and an ampicillin resistance gene (*ampR*) for propagation and selection in *Escherichia coli*. Both plasmids also contain a 1,974bp BamHI DNA cassette harboring the *Mccp dnaA* gene surrounded by its intergenic regions for replication in *Mccp* cells, as well as a 4,034bp PstI DNA cassette originating from the pIVT-1 with a tetracycline resistance gene (*tet*(M)) for mycoplasma selection. PMccpO1 differs from pMccpO2 by the orientation of the BamHI DNA cassette. Plasmid maps were created using SnapGene.

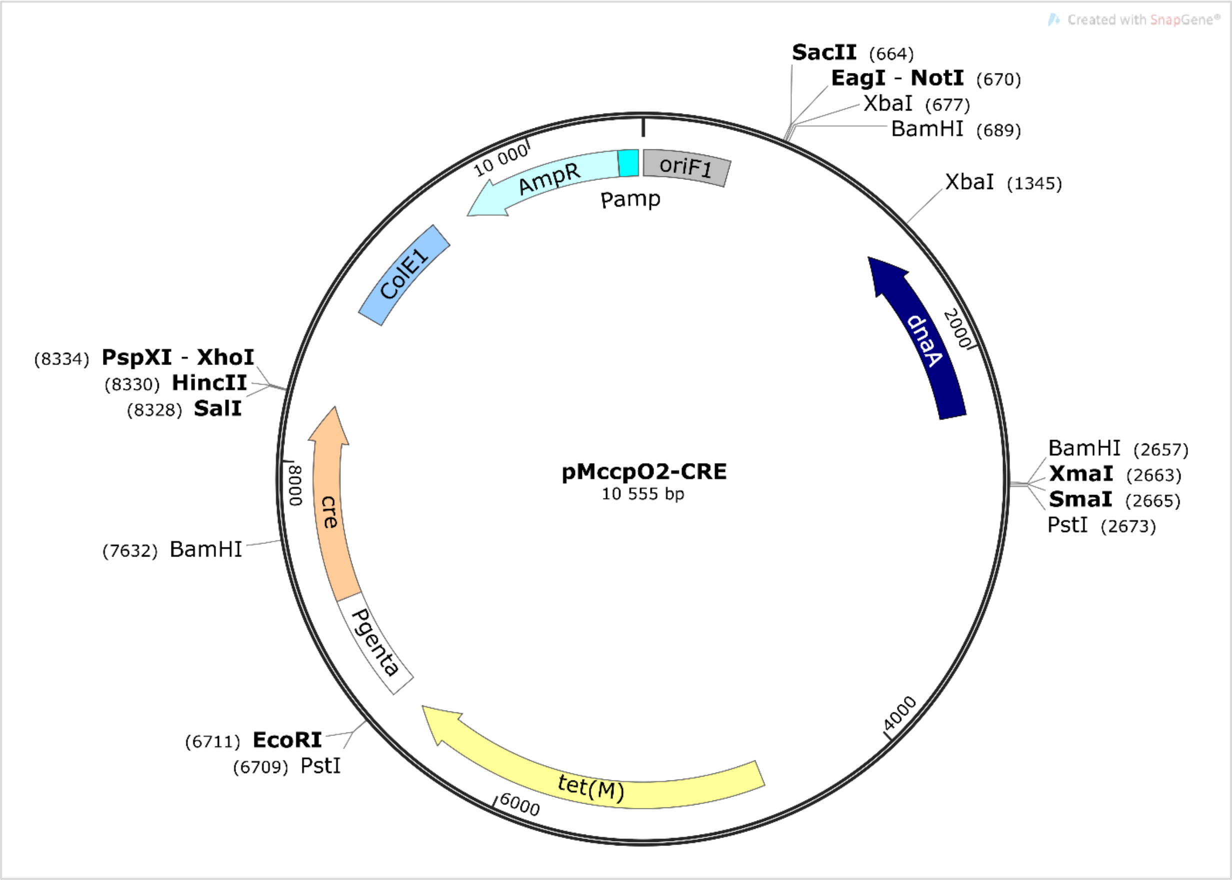

Figure S4. Map of the *OriC* replicative plasmid pMccpO2-CRE. This plasmid derived from plasmid pMccpO2, described in Figure S3. It harbors the *cre* gene under the gentamicin promoter. The plasmid map was created using SnapGene.

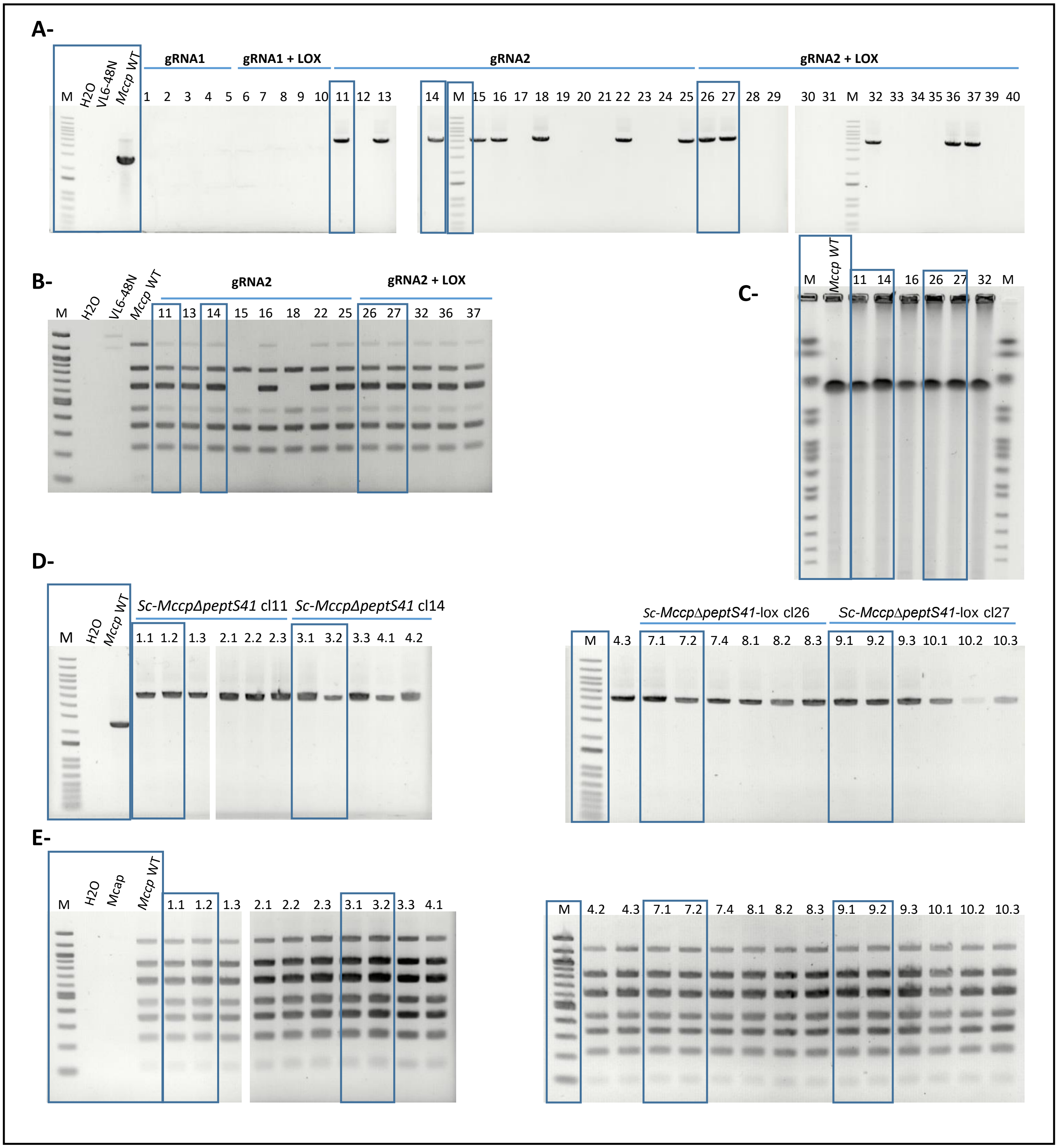

Figure S5: Full images used to produce the panels in Figure 2. The sections spliced together are highlighted with blue rectangles.

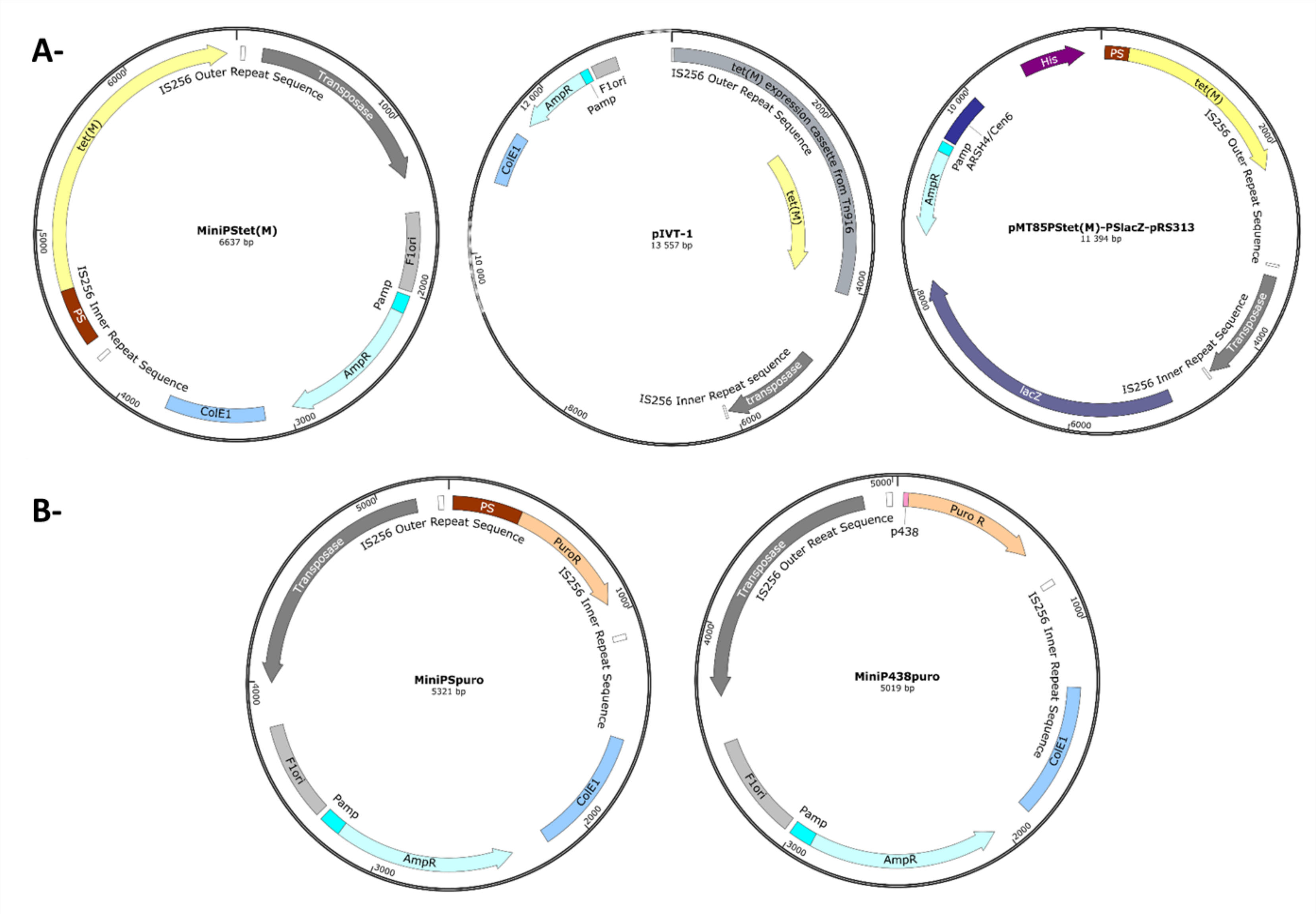

Figure S6. Map of plasmids MiniPS*tet(M)*[3], pIVT-1[4], pMT85PS*tet(M)*-PS*lacZ*-pRS313[1], MiniPS*puro*[3] and MiniP438*puro*. (A) Plasmids carrying a tetracycline resistance marker. (B) Plasmids carrying a puromycin resistance marker. MiniP438*puro* is the exact copy of MiniPS*puro* except for the P438 promoter[5] in place of the spiralin promoter (PS). Plasmid maps were created using SnapGene.

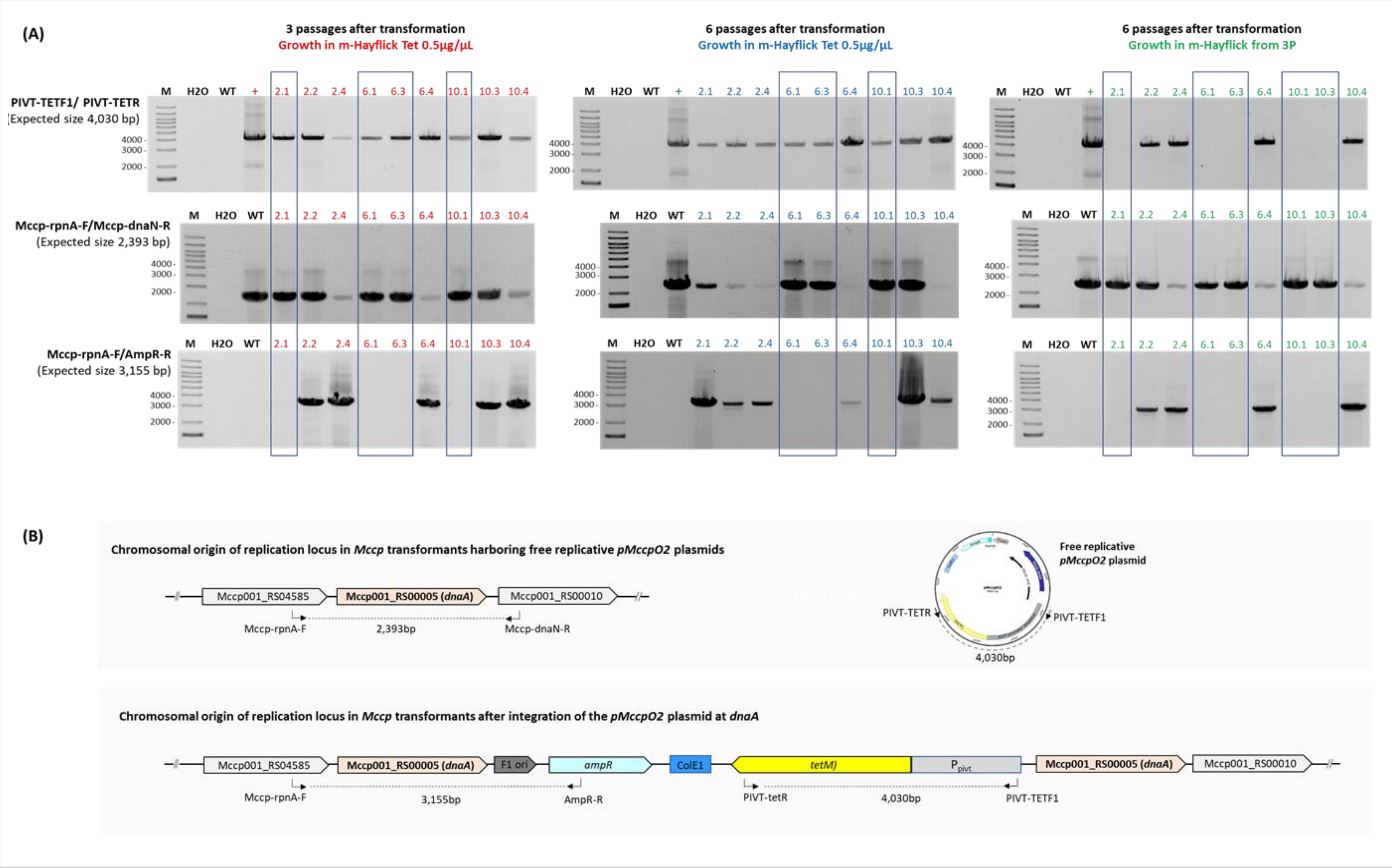

Figure S7. See legend on next page

Figure S7. Detection of homologous pMccpO2 *oriC* plasmids in *Mccp* transformants and state of integration over time. (A) Genotypic analysis of *Mccp* transformants by PCR. M": 1Kb plus DNA Ladder (ThermoFisher); "H2O": negative control without DNA; "WT": Wild-type *Mccp* strain Abomsa gDNA; "+": purified pMccpO2 plasmid; **(B)** Top panel: schematic representation of the chromosomal origin of replication region in *Mccp* transformants harboring free pMccpO2 plasmids; Bottom panel: schematic representation of the chromosomal origin of replication region in *Mccp* transformants after integration of pMccpO2 at *dnaA*.

This figure documents the presence and behavior of pMccpO2 plasmids in *Mccp* transformants during cell propagation. More specifically, the integration status of pMccpO2 plasmids was studied by PCR using 3 pairs of primers after 3 and 6 passages in either selective or non-selective broth. One passage corresponds to the transfer of 10 µL of mycoplasma culture to 1 mL of medium (1/100 dilution) followed by an incubation period of ≥24h at 37 °C.
A total of 9 clones from 3 independent transformation experiments (3 per experiment) were tested and the primer pairs used for the analysis were as follows:

- PIVT-TETF1/ PIVT-TETR : this primer pair (specific of the *tet(M)* resistance cassette) enables specific amplification of a 4,030 bp DNA fragment in *Mccp* cells carrying either extrachromosomal or genome-integrated pMccpO2 molecules (part B, top and bottom panels).

- Mccp-rpnA-F/Mccp-dnaN-R : this primer pair enables specific amplification of a 2,393-bp DNA fragment encompassing the *Mccp dnaA* gene in *Mccp* WT cells and in *Mccp* cells carrying extrachromosomal pMccpO2 molecules (part B, Top panel).

- Mccp-rpnA-F/AmpR-R : this primer pair enables specific amplification of a 3,155-bp DNA fragment in *Mccp* cells that have integrated the pMccpO2 plasmid at the chromosomal dnaA gene by homologous recombination via simple crossing over (part B, Bottom panel).

To be more precise, tetracycline-resistant colonies were picked in 1mL of m-Hayflick medium containing 0.5µg/µL tetracycline and subcultured for a total of 6 passages. The first 3 passages were performed in m-Hayflick medium containing 0.5µg/µL tetracycline (part A, red column) while passages 4 to 6 were carried out either in the presence (part A, blue column) or absence (part A, green column) of tetracycline.

After 3 passages in m-Hayflick plus tetracycline (part A, red column), all clones showed a 4,030bp-*tet(M)* amplicon (gel at the top), indicating that they all possessed the pMccpO2 plasmid. Then, PCR performed with primer pairs Mccp-rpnA-F/Mccp-dnaN-R and Mccp-rpnA-F/AmpR-R (which discriminate between clones presenting the plasmid either free or integrated into the chromosome) revealed 2 types of PCR profile:

➔ The first profile (cl. 2.1, 6.1, 6.3 and 10.1, blue box) showed the presence of an amplicon only with the primer pair Mccp-rpnA-F/Mccp-dnaN-R indicating that the plasmid is free in these clones.
➔ The second profile (cl. 2.2, 2.4, 6.4, 10.3, 10.4) showed the presence of an amplicon with both pairs of primers, suggesting the occurrence of a mixed population of cells, carrying either free or integrated plasmids (or both).

Therefore, after 3 passages in liquid medium with antibiotics, the plasmid was found totally free in 4 of the 9 clones analyzed and in mixture in 5 others. **The finding of extrachromosomal DNA molecules in *Mccp* transformants here clearly demonstrates that the *oriC* region predicted *in silico* for *Mccp* enables the replication of plasmids and that pMccpO2 is a replicative plasmid.**

Then, to monitor the evolution of these clones and plasmid stability over time, 3 additional passages were performed in m-Hayflick medium either with tetracycline (part A, blue column) or without tetracycline (part A, green column).

When the selection pressure was maintained in the culture medium for a further 3 passages (part A, blue column), the PCR profile changed for some clones and not for others compared to 3P:

- ➔ Of the four clones carrying free plasmids at 3P, three (cl. 6.1, 6.3 and 10.1, blue box) maintained an unchanged PCR profile at 6P, meaning that they still carried the plasmid as a free molecule in the cytoplasm after 6 *in vitro* passages and one (cl 2.1) showed a different PCR profile. In this clone, a PCR amplicon was detected with both primer pairs, indicating that the plasmid was free in some cells and integrated at the *oriC* in others.
- ➔ Of the five clones showing a mixed profile at 3P, all again showed a two-band profile at 6P (indicating a mixed population). In three of them (cl2.4, 6.4 and 10.4), the band at 2,393 bp (free plasmid) was barely visible, suggesting that in these clones the majority of the cells had integrated the plasmid at the *oriC*.

In summary, after 6 passages in liquid medium plus tetracycline, the plasmid was found totally free in 3 out of 9 clones and in mixture in the other 6. This experiment showed that when selection pressure is maintained over time, the pMccpO2 plasmid tends to integrate the genome, first generating mixed clones and then pure clones consisting solely of cells in which the plasmid sequence is integrated at the origin of replication.

Interestingly, when the selection pressure was removed from the culture medium from passage 3 onwards (part A, green column), the PCR profile changed for several clones, but not in the same way:

- ➔ Clones carrying plasmids in free form at 3P (cl. 2.1, 6.1, 6.3 and 10.1) lost them all after "tetracycline-free passages", as shown both by the absence of amplicon at 4,030bp with the primer pair PIVT-TETF1/ PIVT-TETR and the presence of amplicon at 2,393bp with the primer pair Mccp-rpnA-F/Mccp-dnaN-R.
- ➔ Finally, among, the 5 clones with a mixed profile at 3P, 4 showed an unchanged profile at 6P (cl. 2.2, 2.4, 6.4, 10.4) and one (cl10.3) appeared to be made up of cells that had lost the plasmid. This suggests that cells with an intact origin of replication and no plasmid to replicate had a growth advantage over the others.

In summary, after 6 passages in liquid medium without antibiotics, pMccpO2 plasmid was found free in 5 out of 9 clones (blue box), and in mixture in the other 4.

These data confirmed that the pMccO2 plasmid is a replicative plasmid, that it can be maintained free in the form of extra-chromosomal molecules in certain clones up to at least 6 passages after transformation and that it can be lost in an antibiotic-free environment.

We concluded from these experiments that we could use the pMccpO2 plasmid to express the heterologous *cre* gene over a few generations, and that it would then be possible to get rid of the pMccpO2-CRE plasmid simply by removing the selection pressure.

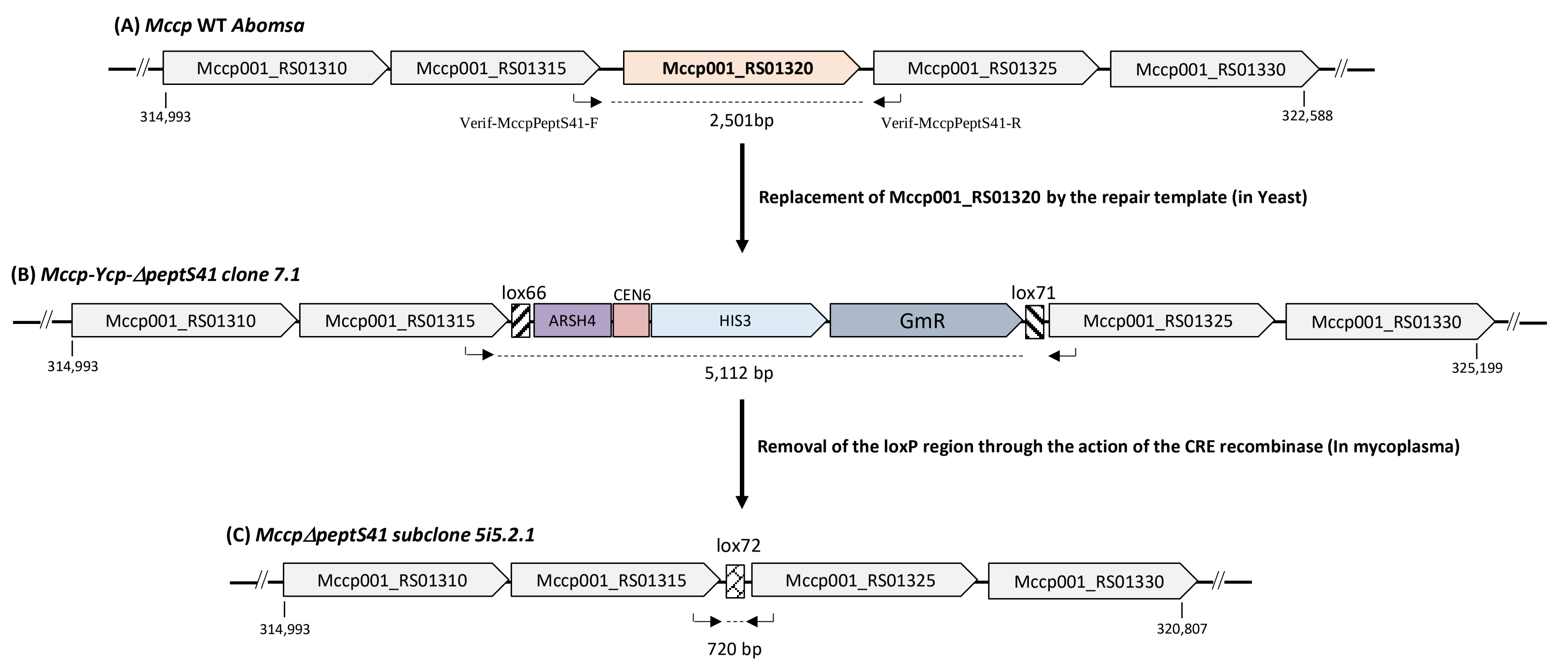

Figure S8: Schematic representations of the Mccp001_RS01320 locus at different steps of the experiment. (A) Mccp001_RS01320 locus in the *Mccp* WT Abomsa genome. **(B)** In-yeast replacement of the *Mccp* target gene (Mccp001_RS01320) by a repair template composed of the yeast elements (ARSH4, CEN6, HIS3), the gentamicin resistance marker and two loxP sites (lox66 and lox71)[2], using the CReasPy-cloning method. The in-yeast modified *Mccp* genome was further transplanted into *McapΔRE* cells and *Mccp* mutants resistant to gentamicin were recovered (*e.g. Mccp-Ycp-ΔpeptS41* clone 7.1). **(C)** Removal of the "loxP region" after CRE/LoxP recombination. In order to obtain an *MccpΔpeptS41* mutant free of any marker, *Mccp-Ycp-ΔpeptS41* clone 7.1 was transformed with the pMccpO2-CRE replicative plasmid to remove all extra DNA sequences added during the genome engineering process, and then passaged several times in m-Hayflick medium without antibiotics to get rid of the replicative plasmid pMccpO2-CRE. A clone free of any marker (Gm^S and *tet*$(M)^S$) was recovered (*MccpΔpeptS41* subclone 5i5.2.1). Small black arrows: PCR primers Verif-MccpPeptS41-F/R used for PCR screening (**Table S1**); Expected amplicon sizes are given for each case.

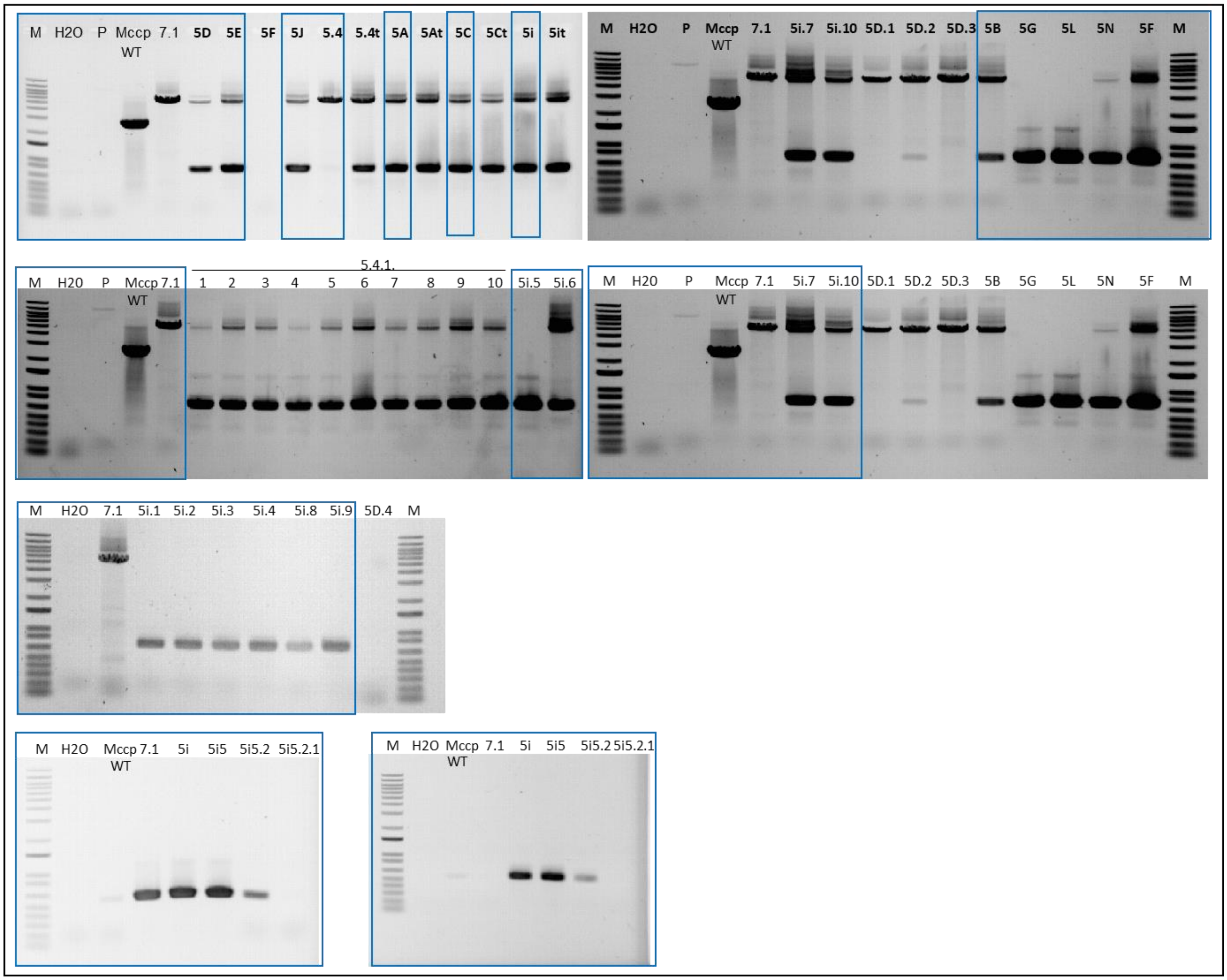

Figure S9: Full images used to produce the panels in Figure 4. The sections spliced together are highlighted with blue rectangles.

Table S1: Primers and Plasmids (see Excel document)

Table S2: Transformation of *Mccp* with plasmids carrying an antibiotic resistance cassette to either tetracycline or puromycin

Plasmid (10 µg)	Number of colonies on selective plates*	Transformation efficiencies (Number tfs CFU/total CFU/µg of plasmids)	Number of colonies picked
**1- MiniP438*puro*****	**55**	**-**	**-**
**2- MiniPS*puro*****	**139**	**-**	**4**
**3- pIVT-1**	**146**	**2.26×10^{-9}**	**6**
**4- MiniPS*tet(M)***	**0**	**-**	**-**
**5-pMT85PS*tet(M)*-PS*lacZ*-PRS313*****	**12***	**$1,85 \times 10^{-10}$*****	**3**
**6-pMT85P*genta*-PS*lacZ*-PRS313******	**213**	**3.3×10^{-9}**	**-**
**7 - No DNA (Tet 0.5 µg.mL-1)**	**0**	**-**	**-**
**8 - No DNA (Puro 8 µg.mL-1)**	**66**	**-**	**-**
**9 - No DNA (Genta 300 µg.mL-1)**	**0**	**-**	**-**

* Transformed cells were seeded on m-Hayflick selective plates directly after transformation
** Transformation efficiencies were not calculated for assays performed with plasmids carrying a puromycin resistance cassette, because genotypic analyses showed that the colonies that appeared on the plates containing 8 µg.mL-1 of puromycin were all spontaneous mutants.
****Mccp* transformation experiment with plasmid pMT85PS*tet(M)*-PS*lacZ*-PRS313 was carried out in an independent experiment. During that experiment, the transformation efficiency with pIVT-1 (used as a positive control) was equal to 1.3×10^{-9} transformants CFU/total CFU/µg of plasmids and that of pMT85PS*tet(M)*-PSlacZ-PRS313 was equal to 1.85×10^{-10} transformants CFU/total CFU/µg of plasmids, *i.e.* around 10 times lower.
**** Plasmid used as positive control.

Table S3: SNP-Calling (see Excel document)

REFERENCES

1. Labroussaa, F. *et al*. Impact of donor-recipient phylogenetic distance on bacterial genome transplantation. *Nucleic Acids Research* **44**, 8501–8511 (2016).
2. Shaw, D., Serrano, L. & Lluch-Senar, M. Lox'd in translation: Contradictions in the nomenclature surrounding common lox-site mutants and their implications in experiments. *Microbiology (United Kingdom)* **167**, 1–11 (2021).
3. Algire, M. A. *et al*. New selectable marker for manipulating the simple genomes of Mycoplasma species. *Antimicrobial agents and chemotherapy* **53**, 4429–4432 (2009).
4. Dybvig, K., French, C. T. & Voelker, L. R. L. Construction and use of derivatives of transposon Tn4001 that function in Mycoplasma pulmonis and Mycoplasma arthritidis. *Journal of bacteriology* **182**, 4343–4347 (2000).
5. Pich, O. Q., Burgos, R., Planell, R., Querol, E. & Piñol, J. Comparative analysis of antibiotic resistance gene markers in Mycoplasma genitalium: Application to studies of the minimal gene complement. *Microbiology* **152**, 519–527 (2006).
